# Impact of increasing morphological information by micro-CT scanning on the phylogenetic placement of Darwin wasps (Hymenoptera, Ichneumonidae) in amber

**DOI:** 10.1186/s13358-023-00294-2

**Published:** 2023-11-03

**Authors:** Alexandra Viertler, Karin Urfer, Georg Schulz, Seraina Klopfstein, Tamara Spasojevic

**Affiliations:** 1 Natural History Museum Basel, Augustinergasse 2, 4051 Basel, Switzerland; 2 Institute of Ecology and Evolution, University of Bern, Baltzerstrasse 6, 3012 Bern, Switzerland; 3 Natural History Museum St. Gallen, Rorschacher Strasse 263, 9016 St. Gallen, Switzerland; 4 Core Facility Micro- and Nanotomography, Department of Biomedical Engineering, University of Basel, Hegenheimermattweg 167 B/C, 4123 Allschwil, Switzerland; 5 Biomaterials Science Center, Department of Biomedical Engineering, University of Basel, Hegenheimermattweg 167 B/C, 4123 Allschwil, Switzerland

**Keywords:** Fossils, RoguePlots, Parasitoids, Baltic Amber, Dominican Amber, Bayesian phylogenetic inference

## Abstract

The correct interpretation of fossils and their reliable taxonomic placements are fundamental for understanding the evolutionary history of biodiversity. Amber inclusions often preserve more morphological information than compression fossils, but are often partially hidden or distorted, which can impede taxonomic identification. Here, we studied four new fossil species of Darwin wasps from Baltic and Dominican amber, using micro computed tomography (micro-CT) scans and 3D reconstructions to accurately interpret and increase the availability of morphological information. We then infer their taxonomic placement in a Bayesian phylogenetic analysis by combining morphological and molecular data of extant and fossil Darwin wasps and evaluate the impact and usefulness of the additional information from micro-CT scanning. The results show that although we gained significant morphological information from micro-CT scanning, especially concerning measurements and hidden dorsal and ventral structures, this did not impact subfamily-level placement for any of the four fossils. However, micro-CT scanning improved the precision of fossil placements at the genus level, which might be key in future dating and diversification analyses. Finally, we describe the four new fossil species as *Rhyssa gulliveri* sp. nov. in Rhyssinae*, Triclistus levii* sp. nov. in Metopiinae, *Firkantus freddykruegeri* gen. et. sp. nov. in Pimplinae and *Magnocula sarcophaga* gen. et sp. nov. in Phygadeuontinae. The first two species are the first known representatives of the subfamilies Rhyssinae and Metopiinae in amber.

**Supplementary Information:**

The online version contains supplementary material available at 10.1186/s13358-023-00294-2.

## Introduction

Fossils play a significant role in our understanding of the history of life on Earth. By looking at the fossil record, we gain valuable insights, for example, about diversification processes (Nicholson et al., 2014), the past diversity of species (Bush & Bambach, 2011), trait evolution (Richter et al., 2022; Wilson et al., 2015), and interactions of phenotypes and environment (Leung, 2017; Wilson et al., 2015). To profit fully from fossil evidence, many analyses rely heavily on the accurate interpretation of the morphological characteristics of a fossil, often in the form of its correct taxonomic classification (Sansom, 2014; Sansom & Wills, 2013; Wiens & Moen, 2008). The interpretation of a fossil’s morphology depends intensely on the medium in which the fossil is embedded and its taphonomic history, and thus its preservation.

Amber, a fossilized tree resin, is a potent medium to preserve small organisms like insects in three dimensions. The preservation of amber fossils is influenced by many factors before, during and after the amber-forming processes; for instance, consecutive amber flows can create layers in the amber that distort the light, and weathering often leads to opaqueness or darkening of the amber (Martínez-Delclòs et al., 2003; Poinar & Mastalerz, 2000). This results in some body parts of an amber specimen being invisible, which is particularly unfortunate when they carry key traits for identifying a taxon. Missing data or misinterpretation of characters which are present as absent (e.g., due to taphonomic processes) might lead to a fossil appearing to be more ancestral than it actually is (Sansom, 2014; Sansom & Wills, 2013). The opposite is observed when morphological characters that appear absent are cautiously treated as “unknown”, which could make a fossil resemble more extant taxa (Sansom, 2014). The wrongly interpreted fossils might lead to several problems in understanding the evolutionary history of a group, such as biased estimates of its time of origin or the timing of major diversification events.

To correctly interpret and increase the amount of morphological information from amber specimens, non-destructive micro-CT scanning is an increasingly popular technique (Racicot, 2017; Sutton et al., 2014). Often micro-CT scanning reveals obscured external characters that are necessary for identification and comparison with extant taxa, as for male pedipalps in spiders (Penney et al. 2007) or number of antennal segments in Strepsiptera (Henderickx et al. 2013). And while insects in amber are often disintegrated, it is not rare that internal anatomical structures are preserved (van de Kamp et al., 2014), which can be revealed with micro-CT scanning as reported in digestive tube in termites (Coty et al., 2014), genital features in flies (Kehlmaier et al., 2014) and beetles (Perreau & Tafforeau, 2011), etc.

Morphological information revealed by micro-CT scanning can improve and enrich species descriptions (Henderickx et al., 2013; Kundrata et al., 2020), or it can be used in a phylogenetic analysis to infer the position of a fossil taxon relative to other fossil or to extant taxa. The latter has been done in morphology-only analyses (e.g., Garwood et al. 2017; Klopfstein & Spasojevic, 2019; Moser et al., 2021) as well as in combined-evidence analyses (Fikáček et al., 2020; Qvarnström et al., 2021; Spasojevic et al., 2021), where molecular data are used to stabilize the relationships among extant taxa. If the phylogenetic analyses are conducted in a Bayesian framework, then phylogenetic uncertainty is accounted for, which allows for a more objective consideration of fossil placement (Klopfstein & Spasojevic, 2019). However, the impact of adding morphological information gained by micro-CT scanning on the taxonomic placement of amber fossils has to our knowledge never been systematically investigated. An especially interesting group for analyzing the improvement of fossil placement with micro-CT scanning is the largest hymenopteran family, the Darwin wasps (Ichneumonidae) (Klopfstein et al., 2019). They are morphologically very diverse, with 47 subfamilies, including five fossil subfamilies (Broad et al., 2018), and often share plesiomorphic traits. When comparing the extant diversity of over 25,000 described species (Yu et al., 2016) to the extinct species number, which is only little over 300 described fossil species, it becomes clear how poorly the ichneumonid fossil record is studied. Of those described fossil species, only 45 are found in amber, 22 of which in Baltic amber (Manukyan, 2023; Manukyan & Zhindarev, 2021), eight in Myanmar amber (Kopylov et al., 2021; Li et al. 2017), seven in Taimyr amber (Kopylov, 2012; Townes, 1973), and three in both French Oise amber (Viertler et al., 2022b) and Canadian amber (McKellar et al., 2013).

We here study four fossil Darwin wasps in amber, three from Baltic and one from Dominican amber, and investigate the impact of 3D reconstructions from micro-CT scans on the precision of their phylogenetic placement. The four chosen specimens are all sufficiently visible, under a standard microscope for confident placement in Ichneumonidae, while showing varying degrees of visibility otherwise due to air bubbles, irregularities in the amber or other inclusions. Based on a large dataset containing morphological and molecular information of both extant and fossil Darwin wasps, which we complemented for our purpose, we conduct a Bayesian combined-evidence analysis and compare the phylogenetic placement of the fossils before and after adding the micro-CT scanning data, which in all cases revealed additional morphological details. Based on the inferred phylogenetic placement, we place all four fossils in extant ichneumonid subfamilies. All four fossils are described as new species of which two represent the first representatives of their respective subfamilies in Baltic amber, and one the first clear Darwin wasp from Dominican amber.

## Materials and methods

### Fossil specimens

To test for the change in phylogenetic placement with increased morphological details, we chose four undescribed amber fossils from two insect-rich deposits, Dominican amber (one fossil) and Baltic amber (three fossils), with varying quality of preservation (see under “Type condition” in the “Systematic placement” section).

The fossil found in Dominican amber (#DO_3441-M) is deposited at the Staatliches Museum für Naturkunde Stuttgart (Germany). The exact locality of the excavation of this specimen is unknown. Two of our three Baltic amber inclusions (#NHMD_876111, #NHMD_876130) were collected on the West Coast of Jutland (Denmark), towards the North Sea (pers. comm. L. Vilhelmsen 2023) and belong to the Natural History Museum in Copenhagen (Denmark). The third Baltic amber piece (NMB F3742) is from the Kaliningrad region, from the amber mine Yantarny in Russia, and is deposited in the collection of the Natural History Museum in Basel (Switzerland). The exact origin of Kaliningrad amber is difficult to assess, since it is collected from different localities and stratigraphic layers and only rarely exactly labelled (Bukejs et al., 2020).

### Micro-CT scanning

To maximise morphological information from our four fossil specimens, we performed microtomography scans at the Core Facility “Micro- and Nanotomography” of the University of Basel (Switzerland) using a phoenix|x-ray nanotom® m (Waygate Technologies Wunstorf, Germany). The micro-CT scan settings for each fossil are in Table 1. Subsequently, the micro-CT scan data were segmented in AVIZO^Ⓡ^ 7.0 software (Visualization Sciences Group). The scans of all four fossil species are available on MorphoBank (http://morphobank.org/permalink/?P4882).

**Table 1:** Micro-CT scan settings for each fossil specimen

Fossil specimen	NMB F3742	#DO_3441-M	#NHMD_876111	#NHMD_876130
Acceleration voltage	70 kV	60 kV	70 kV	60 kV
Beam current	260 µA	310 µA	260 µA	310 µA
Projections over 360°	1440	1440	1440	1000
Pixel size	7.5 µm	4.0 µm	7.0 µm	2.2 µm
Exposure time at each rotation	3 s	4 s	3 s	6 s

### Morphological terminology and descriptions

The terminology primarily follows Broad et al. (2019), except wing venation, which follows Spasojevic et al., (2018) with the adjustment of cell 1 + 2Rs being named the “areolet”. The areolet is called “closed” when vein 3rs-m is present, or “open” when it is absent. The short vein 1Rs + M is referred to as the “ramulus”. Tergites and sternites are numbered in the text and abbreviated as “T1”, “T2”, etc., and “S1”, “S2”, etc., respectively.

Measurements for the descriptions were made from the 3D reconstruction in MeshLab (Cignoni et al., 2008). For the metasoma length, we added up the dorsal lengths of each tergite, starting at the base of T1 until the apex of the metasoma, without the ovipositor. If a measurement is given as a range, this indicates that the right and left body side slightly differed from each other when measured. When antennal segments are given as a range, the interpretation of some segments was uncertain. Photos were taken with a Keyence VHX 600 camera system (see Keyence, 2023) with a magnification of 50–200 using stacking techniques. For the photos, the amber pieces were mounted between two glass slides using glycerine (99.5%) as a medium between the slide and amber piece. The interpretative drawings of the fossils were made in Adobe Photoshop (vers. 25.0.0), using different photos and angles from the micro-CT scan of the specimens as templates. Uncertain interpretations of morphological structures are indicated by dotted lines, and shading with straight faint lines emphasises depth of some structures.

Specific morphological characters that were used to compare the fossils with subfamilies and genera or species are taken from various references (Broad et al., 2018; P. Kasparyan, 1994; Townes, 1969a, 1969b, 1970, 1971).

To express uncertainty in the fossil classification, we follow the open nomenclature framework (Matthews, 1973), where a question mark is used after the subfamily name to denote uncertain affiliation.

### Morphological matrix

For the phylogenetic analyses, we used the morphological matrix of Spasojevic et al., (2021), modified to fit our investigated fossils. We first identified candidate subfamilies for our fossil specimens based on preliminary character evidence, and then adjusted the taxon and character sampling (Additional files 1, 2 and 3). The final morphological matrix consists of 226 characters, 67 of which are binary and 159 are multistate (Additional file 4). From the latter, 15 characters represent measurement ratios, which were originally recorded as continuous and subsequently transformed into discrete, six-state characters using the gap weighting approach described in Thiele (1993), as MrBayes (Ronquist et al. et al., 2012) only allows up to six ordered states (see below).

To assess the impact of increasing morphological information on the placement of fossils, we created two morphological matrices. The first matrix contains the four new fossil specimens coded before the micro-CT scan, where all inaccessible characters were coded as “?”. Here we only included measurements (ratios) when they were directly measurable in one plane. When dimensions are not directly measured, but described in a state (e.g., 1.5–2 × longer than wide), and not in one plane, they were either coded as “?” or polymorphic. The second matrix contains all available morphological character information from the 3D models, including all measurements.

### Molecular matrix

We used the molecular dataset from Spasojevic et al., (2021), which consists of the mitochondrial cytochrome oxidase I (COI) gene and 10 nuclear genes [28S, carbamoyl phosphate synthase domain (CAD) and eight other protein-coding genes established for ichneumonids in (Klopfstein et al., 2018)] for 131 taxa for which we also had morphological data. We added sequences for 26 species belonging to the identified candidate subfamilies from GenBank (Additional file 1) and sequenced 10 additional Metopiinae specimens, because we deemed the representation of molecular data for this subfamily too sparse (for protocol see Additional file 5). If sequences were not available for the same species for which we coded the morphology, we used sequences of congeneric species.

### Combined evidence analysis

We performed two main phylogenetic analyses, where we combined molecular data (6280 bp) and either the morphological matrix with or without micro-CT scanning data. The datasets consisted of 199 taxa in total, with all taxa having at least some morphological data. As an outgroup, we used four species of Xoridinae, the sister to the remaining ichneumonid subfamilies (Klopfstein et al., 2018). Non-applicable morphological characters or nucleotide gaps were treated as missing data.

To avoid extensive model testing, we used the morphology model chosen as the best-fitting among four competing extensions of the Mkv model (Lewis, 2001) for a previous version of this morphological matrix (Klopfstein & Spasojevic, 2019). This model assumes equal transition probabilities between the states, coding of only variable characters (ascertainment bias), among-character rate variation and ordering of the originally continuous characters or discrete characters where we can assume stepwise evolution in a specific direction. In our morphological matrices, 58 characters were identified a priori as ordered, with the remaining 169 characters treated as unordered.

We partitioned the concatenated alignment of all genes by gene and codon position and used PartitionFinder (Lanfear et al., 2016) with PhyML (Guindon et al., 2010) to combine similarly evolving partitions (settings: branch lengths = linked; models = all; model selection = BIC, search = greedy (Lanfear et al., 2012)). This resulted in 15 separate partitions. Instead of fixing the preferred substitution model for each partition, we let MrBayes integrate over the entire GTR-model space using reversible-jump MCMC (Huelsenbeck et al., 2004). For each partition, we added gamma-distributed rate variation (+ G) and an invariant sites (+ I) model, according to the results of the PartitionFinder analysis.

The combined-evidence analyses ran initially with four independent runs for 100 million generations and trees were sampled every 1000 generations. Of the four runs, two of them got stuck on tree islands of inferior likelihood, so we excluded them and summarised the two remaining runs. The MCMC convergence of both runs was assessed by the average standard deviation of split frequencies (ASDSF < 0.01), the effective sample size (ESS > 200), the potential scale reduction factor (PSRF < 1.005) and by visualising the model parameters in Tracer. However, even after 100 million generations, some parameter estimates, especially for the COI partitions, did not converge to the set values in both analyses (before and after the CT-scan). Given the complexity of our analysis, including the use of fossils, we deemed the ASDSF < 0.03, the average ESS value > 100 and the PSRF value sometimes > 1.005 satisfactory for the scope of our research question.

We obtained a majority-rule consensus tree with a burn-in of 0.5, both before and after excluding all fossil species. We also produced a phylogeny where all the fossil taxa were included, to assess if our new fossils were attached to any extinct lineages (Additional file 6: Fig. S1). Since this was not the case, we excluded non-target fossils from the consensus tree.

The data matrices and MrBayes command scripts, which include our prior settings on the distribution of model parameters and associated moves, are available in Additional files 7, 8. The phylogenetic analyses were performed using Bayesian inference in MrBayes on the HPC cluster UBELIX of the University in Bern, Switzerland (http://www.id.unibe.ch/hpc).

In addition to the combined analysis, we also ran analyses with only morphological and only molecular data separately to assess the strength of both datasets to resolve the relationships among the included taxa and to detect any conflicting signals in the topology estimation, which could impact the results of the combined-evidence analysis. The settings were as above.

### Assessment of the phylogenetic placement of the four fossils

To illustrate uncertainty in fossil placement in our Bayesian phylogenetic analyses, we randomly selected 1000 trees from the posterior distribution and provided them, together with the consensus tree where fossils are excluded, to the R (ver. 4.0.2, R Core Team, 2020) function create.rogue.plots() of the RoguePlots package (Klopfstein & Spasojevic, 2019), which summarised and illustrated the attachment probability of each of the fossils to branches on the consensus tree. To determine improvement in taxonomic placement of four newly described fossils, we analyzed the differences in the percentage of coded morphological characters and the coding precision (i.e., from polymorph to monomorph coding) before and after the micro-CT scans. The suggested placement of the combined-evidence analysis for each fossil species was then considered in the systematic placement section of the species description.

## Results

### Newly revealed details from micro-CT scans

The micro-CT scans revealed many important characters that were not accessible before and increased the percentage of the coded characters in all four fossils (Table 2, Additional file 4). The greatest difference was observed in the specimen NHMD 876111, where we increased the completeness of the character matrix by 19% and could code 15 polymorph characters as monomorph after the scan. The least change in the completeness of coding was found in DO-3441-M, where 14% of the total characters were added after the scan. In fossil #DO-3441-M, the wings were not scanned unfortunately, which might be a result of the taphonomic preservation of the wings (Sadowski et al. 2021), where the low density difference between the wings and the amber could have affected the reduction of contrast in this piece. An improvement in uncertainty of coding was also observed in NHMD 876130, where the states in eight polymorph characters were reduced to six monomorph, one duomorph (character with more than two states) and one triomorph (character with more than three states). Overall, the improvement in coding was mostly observed in body measurements, and characters coded from dorsal and ventral view as described below.

**Table 2:** Completeness of the morphological matrix before and after the micro-CT scan

Fossil	Reduction of character states (#Nr. characters)	Completeness in coding (%) before / after micro-CT scan	Attachment frequency before / after micro-CT scan
DO-3441-M	From polymorph to monomorph (9)	58/72	0.993/0.996 *Triclistus*
NHMD 876111	From polymorph to monomorph (15)	39/58	0.829/0.841 *Lissopimpla*, *Xanthopimpla*
F02444	From polymorph to monomorph (14)	59/75	0.654/0 leading to all Rhyssinae 0.218/0.972 *Rhyssa* 0.101/ < 0.1 *Epirhyssa*, *Rhysella*, *Triancyra*
NHMD 876130	From polymorph to monomorph (6), duomorph (1) triomorph (1)	54/73	0.383/0.235 *Chirotica* < 0.1/0.321 *Hemiteles* 0.126/0.111 leading to Ichneumoniformes

The observed change in the attachment positions and frequencies of the four new fossil species in RoguePlots are shown. Attachment positions with frequency below 10% are not included

**Measurements:** Body measurements, which are represented as 15 ratios in the morphological matrix, were deemed more accurate and precise when taking them from the 3D model, where, in contrast to the photos, image distortions caused by the amber are not present. In the fossil NHMD 876111, many wing measurements were only accessible with the scan, where the wings were indicated as clear folds on the surface of the scan. In all four fossils, the antennae could only be measured on the scans since they were often bowed or partially hidden between the wings.

**Dorsal view:** The scans proved to be of advantage for the dorsal aspect of structures in our fossils. It was possible to verify the absence or presence of modifications on the frons, between the antennal sockets and the median ocellus, and to define the type of modification (e.g., #DO-3441-M with a raised keel). We also were able to code the shape and completeness of the occipital carina, which was either coded as not applicable (i.e., missing) or polymorphic before. Several additional structures were also clearer in the 3D model, especially the propodeal carination, and the shapes and carination of the first and second tergites, which can easily be veiled by wings or legs.

**Ventral view:** Characteristics that are on the ventral side of the body were frequently only visible in the 3D models. For example, the shape of the mesosternum, and presence/absence and modification of the mesosternal posterior transverse carina could only be accurately coded after the scans were performed. In fossil #NHMD_876130, our only female specimen, we were able to restrict the coded states for the hypopygium, which is only clearly visible in ventral view and can give important insights for classification.

**Others:** Next to the measurements, and increase in details of dorsal and ventral structures, we could also characterize several other details that were hidden by milky coatings, cracks or mantling amber flows, such as the apices of claws and the positions of spiracles, as well as the spiracle shapes.

### Phylogenetic placement

**Fig. 1:**
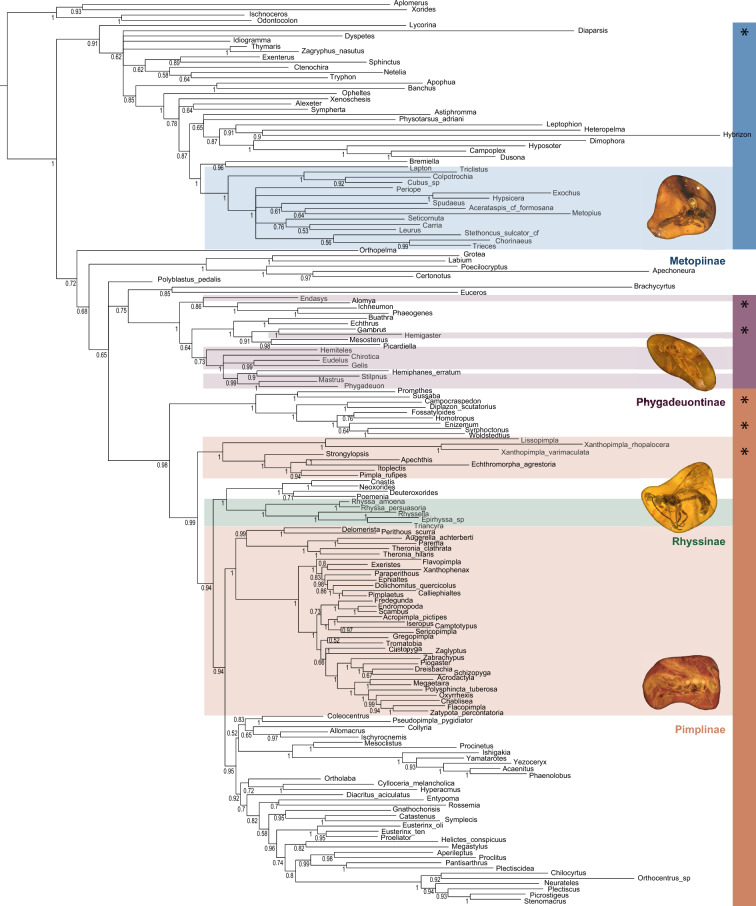
Majority-rule consensus tree from the combined-evidence Bayesian analysis, including 226 morphological characters and 11 molecular genes of 172 extant taxa. Fossils were excluded before building the consensus tree. Coloured boxes denote the respective subfamilies of the fossil taxa, and the bars to the right represent the informal higher groupings (*Ophioniformes, **Ichneumoniformes, ***Pimpliformes). Images of fossil specimens from top to bottom: Do-3441-M, NHMD 876130, F02444, NHMD 876111

The relationships among the subfamilies, including the informal grouping "Pimpliformes", to which two of our target subfamilies (Rhyssinae and Pimplinae) belong, remained similar and well resolved in both our analyses (before and after micro-CT scan) and are in agreement with Spasojevic et al. (2021). Metopiinae was recovered as monophyletic (Fig. 1). *Bremiella* Dalla Torre, 1901 (currently classified in Ctenopelmatinae) and *Lapton* Nees, 1816 (currently classified in Metopiinae) represented a sister group to Metopiinae, which agrees with Alvarado (2018), where the placement of both genera remains unresolved. Three of the four previously suggested clades within Metopiinae (clades 2, 3 and 4 in Alvarado, 2018) were also recovered. The Cryptinae and Phygadeuontinae were not monophyletic, as was expected from previous analyses (Santos, 2017), but both subfamilies were recovered correctly within the informal grouping Ichneumoniformes.

**Fig. 2:**
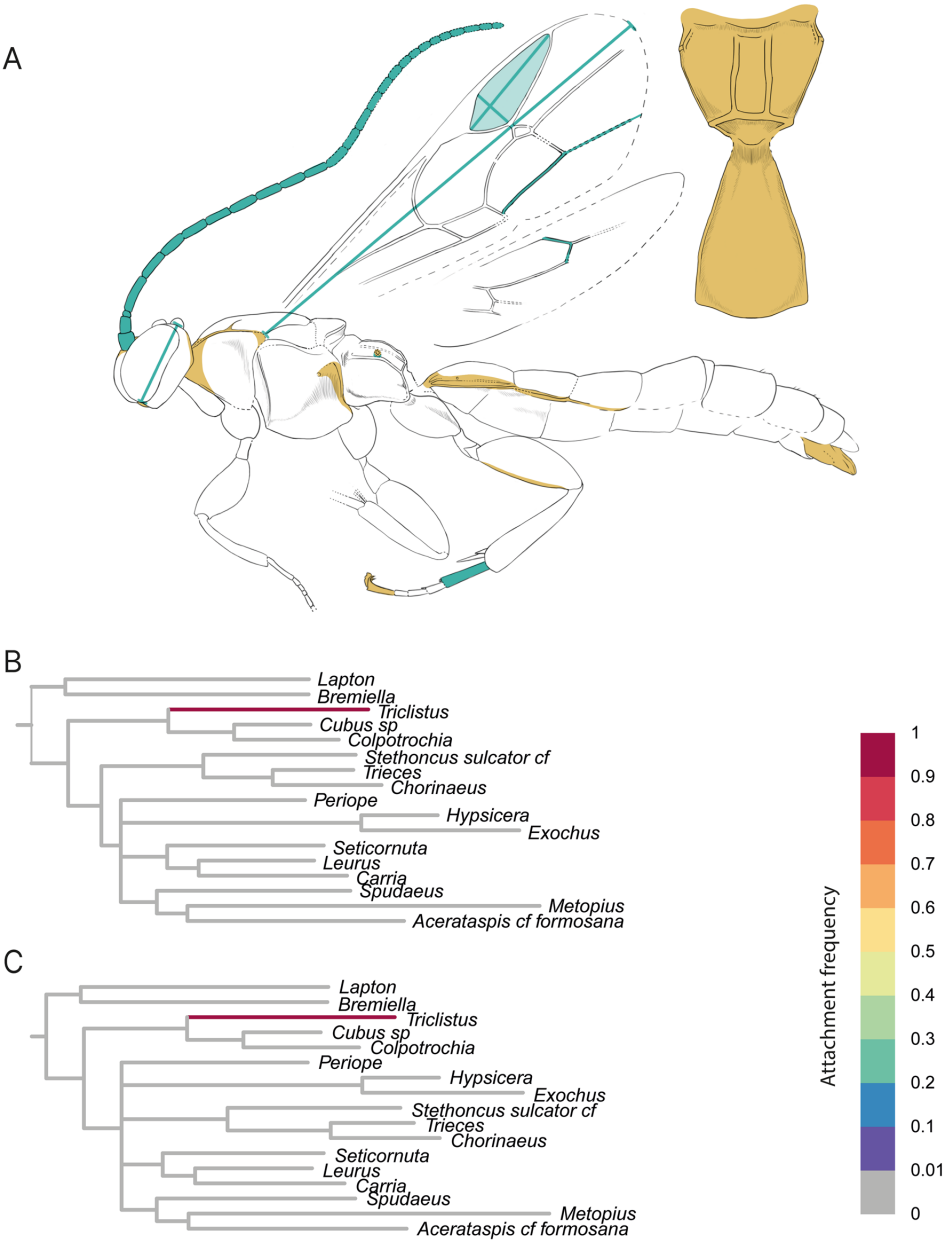
RoguePlot placement of Metopiinae fossil *Triclistus levii* sp. nov. before and after micro-CT scanning. The plots include all branches from the majority-rule consensus tree where the attachment probability was higher than 1%. **A** *Triclistus levii* sp. nov. with colours indicating newly revealed body characteristics after the CT scan. Blue colouration represents newly added measurements; orange highlights either newly coded characters or characters where states could be reduced after the CT scan. **B** Placement before CT scanning. **C** Placement after CT scanning

Regarding the placement of our fossils in the two phylogenetic analyses, fossil DO-3441-M was solely placed in the subfamily Metopiinae. The inferred placement of DO-3441-M did not change with the increase in the number of coded morphological characters (Table 2, Fig. 2), and stayed on the branch leading to the metopiine genus *Triclistus* with a high probability (attachment frequency > 0.99). The additionally coded characters after CT scanning, such as the upper face producing in a triangular process between the antennae, the sclerite covering the upper hind corner of the pronotum, the shape of the propodeal spiracle, the length ratio of veins 4Cu and 5Cu, and the presence of the glymma on T1, were often not only in agreement with *Triclistus*, but also with several other metopiine genera, such as *Colpotrochia* Holmgren, 1856 and *Cubus* Townes & Townes, 1959.

**Fig. 3:**
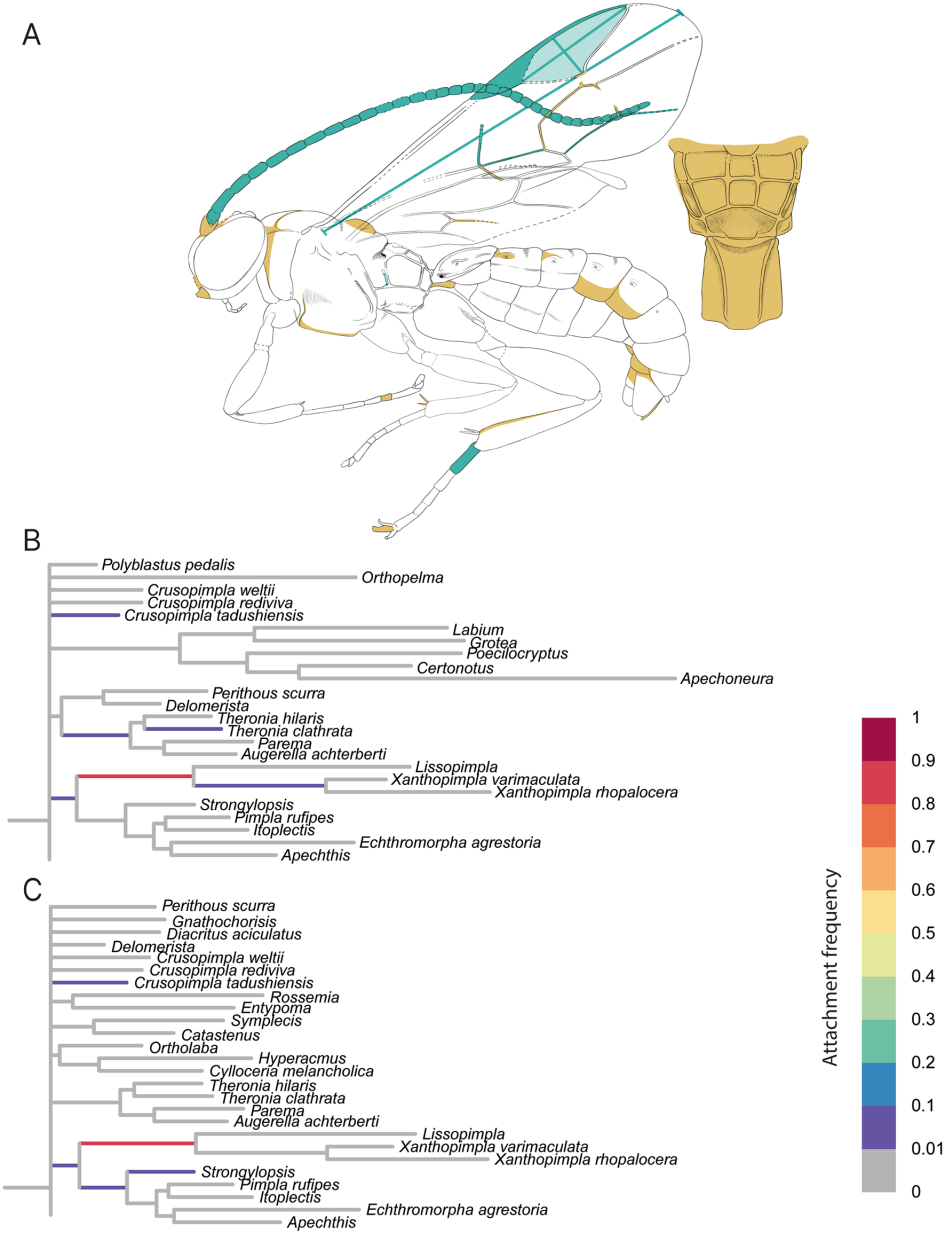
RoguePlot placement of Pimplinae fossil *Firkantus freddykruegeri* gen. et sp. nov. before and after micro-CT scanning. The plots include all branches from the majority-rule consensus tree where the attachment probability was higher than 1%. **A** *Firkantus freddykruegeri* gen. et sp. nov. with colours indicating newly revealed body characteristics. Blue colouration represents newly added measurements; orange highlights either newly coded characters or characters where states could be reduced after the CT scan. **B** Placement before CT scanning. **C** Placement after CT scanning

Fossil NHMD 876111 attached only to branches within Pimplinae (Fig. 3). The highest attachment frequency is reached on the branch leading to the clade of *Lissopimpla* Kriechbaumer, 1889 and *Xanthopimpla* Saussure, 1892 (*X. varimaculata* and *X. rhopalocera*) in the analysis before (0.83) and after (0.84) the micro-CT scan (Table 2). In both analyses, the fossil also attached with frequency below 10% to branches leading to *Crusopimpla tadushiensis*, *Strongylopsis* Brauns, 1896 and to the branch leading to the clade including *Strongylopsis*, *Pimpla rufipes* Miller, 1759, *Itoplectis* Förster, 1869, *Echthromorpha agrestoria* (Swederus, 1787) and *Apechthis* Förster,1869 (Additional files 9, 10).

**Fig. 4:**
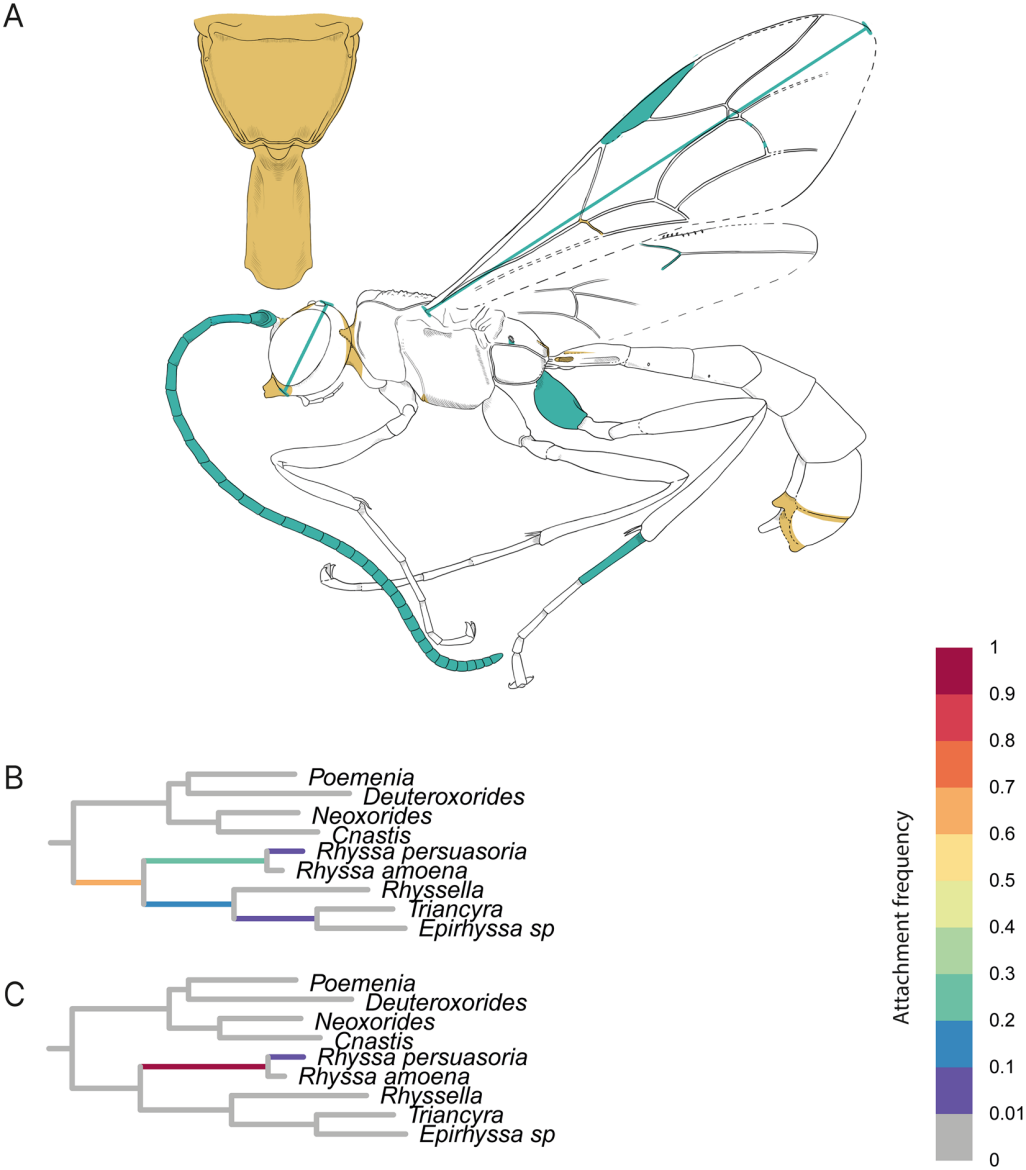
RoguePlot placement of Rhyssinae fossil *Rhyssa gulliveri* sp. nov. before and after micro-CT scanning. The plots include all branches from the majority-rule consensus tree where the attachment probability was higher than 1%. **A** *Rhyssa guliveri* sp. nov. with colours indicating newly revealed body characteristics. Blue colouration represents newly added measurements; orange highlights either newly coded characters or characters where states could be reduced after the CT scan. **B** Placement before CT scanning. **C** Placement after CT scanning

Fossil F02444 solely attached to branches within Rhyssinae (Fig. 4). Before the information from the CT-scan, the highest supported attachment (0.65) of this fossil was on the stem branch of Rhyssinae. After including the additional morphological information, the fossil was recovered within the crown-group Rhyssinae, clearly attached to the branch leading to two included *Rhyssa* species (0.97). The characters that strengthened the attachment to *Rhyssa*, either newly coded or for which we reduced the number of possible states with micro-CT data, are the shape of the occipital carina, the posterior part of the pronotum, the nervulus position in the forewing, and the clear presence of the glymma on T1.

**Fig. 5:**
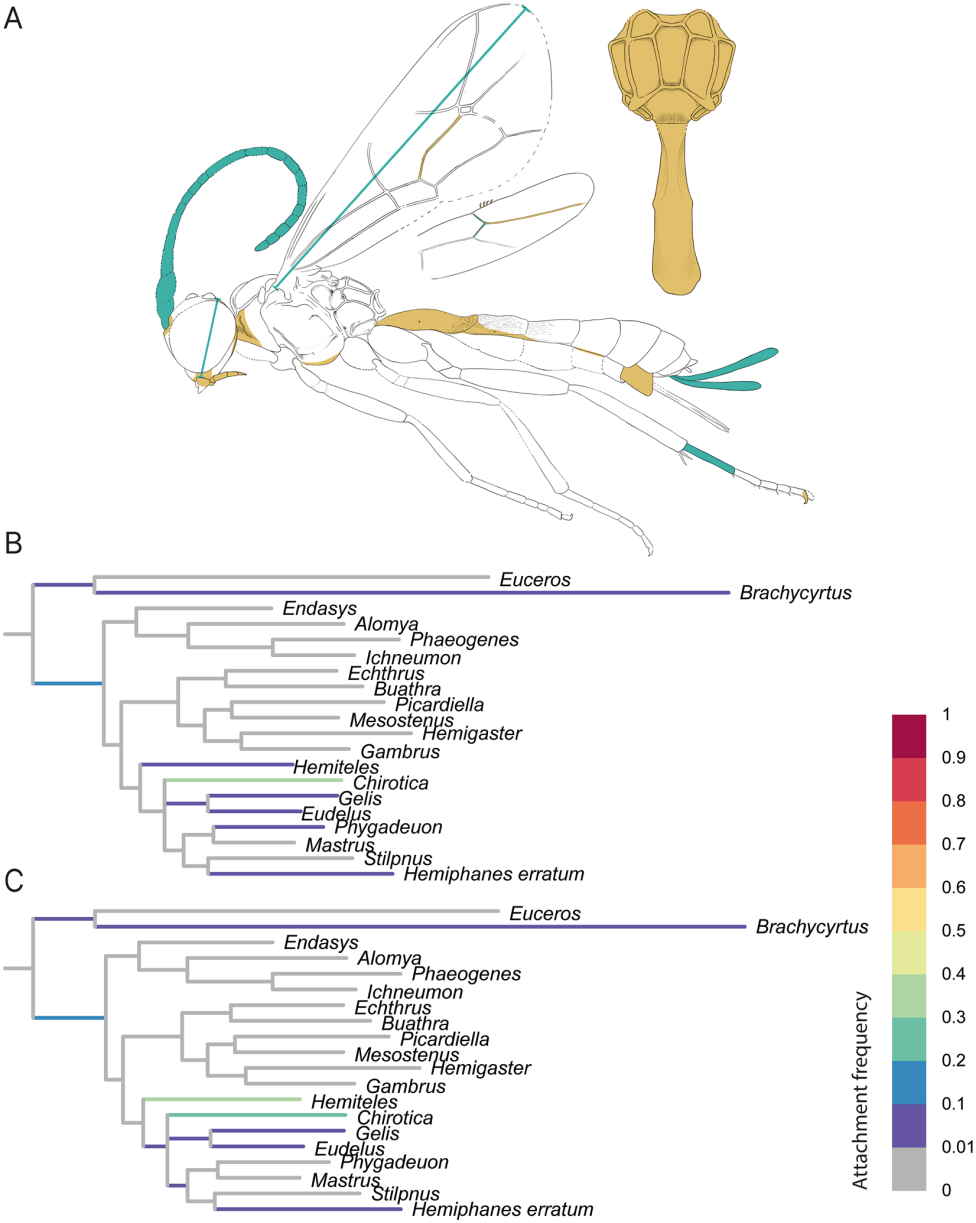
RoguePlot placement of Phygadeuontinae fossil *Magnocula sarcophaga* gen. et sp. nov. before and after micro-CT scanning. The plots include all branches from the majority-rule consensus tree where the attachment probability was higher than 1%. **A** *Magnocula sarcophaga* gen. et sp. nov. with colours indicating newly revealed body characteristics. Blue colouration represents newly added measurements; orange highlights either newly coded characters or characters where states could be reduced after the CT scan. **B** Placement before CT scanning. **C** Placement after CT scanning

Fossil NHMD 876130 was mainly recovered in Ichneumoniformes before micro-CT scanning (Fig. 5), with the exception of some low attachment (< 1%) frequencies leading to fossil Pimplinae (*Crusopimpla weltii* Viertler, Spasojevic & Klopstein 2022 and *C. tadushiensis* Kopylov, Spasojevic & Klopfstein, 2018), a basal branch of Labeninae and to *Brachycyrtus* Kriechbaumer, 1880. The fossil attached on the basal branch to the larger group of Ichneumoniformes with a frequency of 0.13, but most attachments were inferred in the subfamily Phygadeuontinae, with the highest frequency (0.38) on the branch leading to *Chirotica* Förster, 1869. After scanning we could add the lateral shape of the clypeus (also the shape of the apical margin), the modification of the epomia, an interrupted posterior transverse carina in front of the mid legs, a rather elongate hind femur, and the lateral and dorsal shape and dimensions of the first tergite. We were also able to reduce the states for the hypopygium shape. With the increased morphological information, there were still many attachments within Phygadeuontinae, and only a few positions changed. The fossil additionally attached to *Hemiteles* (0.32), while still attaching to *Chirotica* (0.26) and the stem branch to Ichneumoniformes (0.11). Since this specimen shows some characteristics, which are rather rare in Phygadeuontinae, we additionally rerun the analysis but coded “?” for the absence/presence of the sternaulus and its position posteriorly, which are typical for this subfamily. However, the results remain similar, and the highest attaching branches stay the same (to *Hemiteles* and *Chirotica*) with only minor differences in the percentage of attachment frequencies.

## Systematic palaeontology

Order **Hymenoptera** Linnaeus, 1758

Family **Ichneumonidae** Latreille, 1802

Subfamily **Metopiinae** Förster, 1869

Genus ***Triclistus*** Förster, 1869

**Fig. 6:**
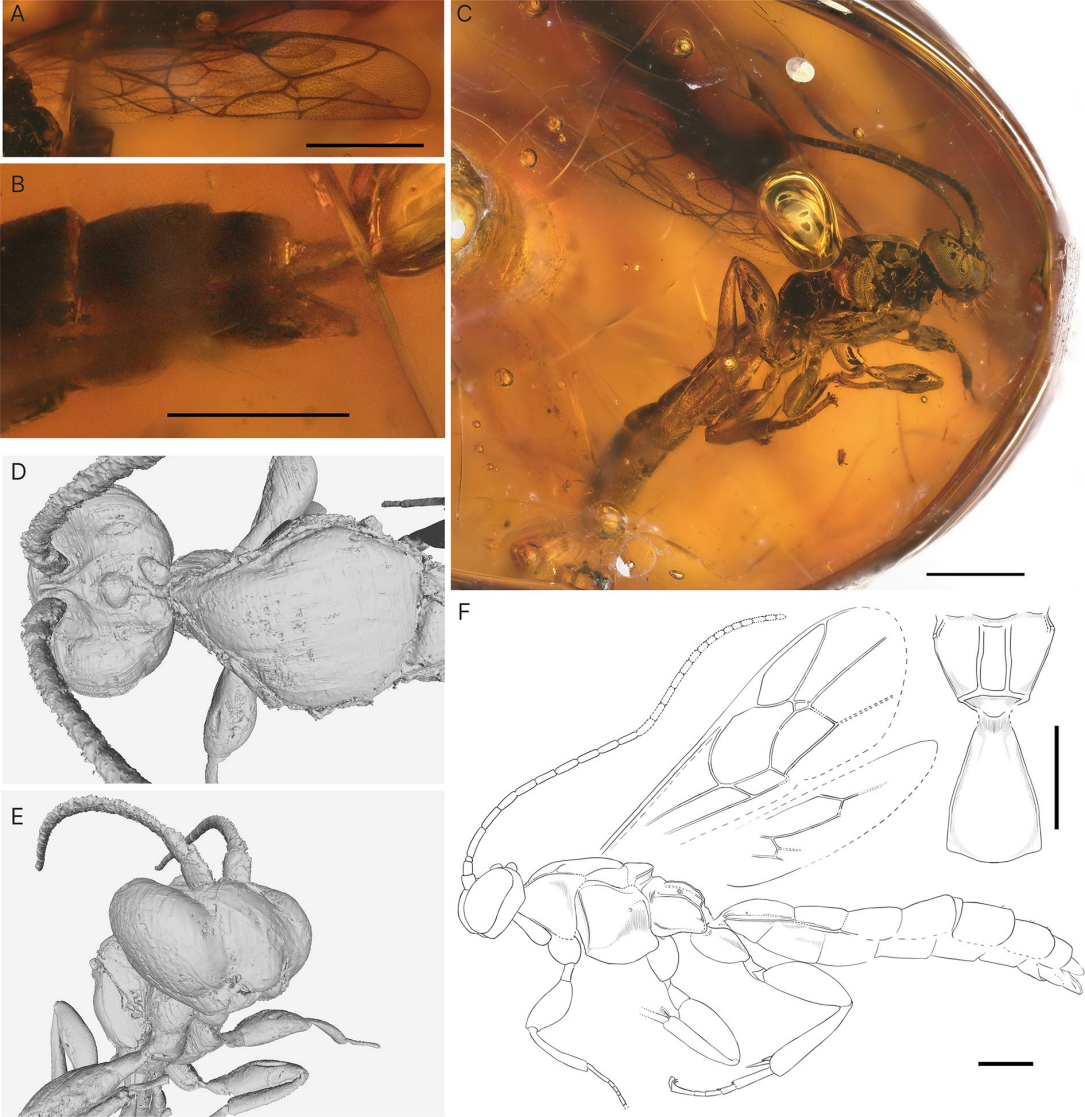
Holotype of *Triclistus levii* sp. nov. **A** Partial fore wing. **B** Metasoma, posterior end with the parameres. **C** Habitus of specimen, lateral view. **D** Head and mesoscutum, dorsal view. **E** Head. **F** Interpretative drawing with an additional drawing of the propodeum and T1, in dorsal view, where photos and micro-CT scan were used as templates. Scale bars A: 1 mm, B: 0.5 mm C: 1 mm F: bottom 1 mm, top right 0.5 mm

***Triclistus levii*** Viertler, Klopfstein & Spasojevic sp. nov. (Fig. 6)

urn:lsid:zoobank.org:act:8EA74EE3-B59E-474D-8F6A-E6A3A01FAA8A

**Etymology**: This species is named in honour of A. Viertler’s loyal fur child Levi.

**Type specimen**: Holotype: male (DO-3441-M). Dominican amber. Location: unknown. Deposited in Germany, Staatliches Museum für Naturkunde in Stuttgart.

**Type condition**: The amber piece is partially darkened, obscuring parts of the fossil, with a drilled hole, made most likely for transforming the amber piece to jewellery. The surface of this amber piece partially cracked due to an accident and was cast in resin to stabilize it for future studies.

**Systematic placement**: The following characters confidently place this fossil in Metopiinae: A convex face without clypeal sulcus, a triangular process on the upper face between the antennal segments, stout and short legs, and shortened tarsal segments. With the interantennal high ridge that reaches to the frons, this fossil resembles *Colpotrochia*, *Cubus*, and *Triclistus* Förster, 1869.

Important characters that support the affinity of the fossil to *Triclistus* were visible before the scanning and include mandible dimensions (Character #1), number of flagellomeres (Character #40), presence of both lateromedian and lateral longitudinal carinae, and posterior transverse carina on the propodeum (Characters #76 & #79), one stout and one long spur on the hind tibia (Character #111), closed areolet with one bulla (Character #128), distally arched 4Rs (Character #137), several wing ratios (e.g. length ratio of Character #144: 2Cu and 1 M + 1Rs and Character #145: 1 M + 1Rs and r-rs), and moderately broad dimension of laterotergite 3 (Character #194). This placement is congruent with the results of the phylogenetic placement.

**Diagnosis**: There are two other fossil *Triclistus* species described, both from sediments, *Triclistus ventrator* (Khalaim, 2008) from the Biamo assemblage (Russia, late Eocene, or Early Oligocene), and *Triclistus bibori* (Viertler et al. 2022a) from the Fur formation (Denmark, Early Eocene).

What stands out in *Triclistus levii* sp. nov. is the length of the basal antennal segments, which are rather long, especially the first segment, which is 4 × longer than posteriorly wide. This was also observed in *T. ventrator*. Nevertheless, *T. levii* sp. nov. differs from *T. ventrator* in having a straight 2m-cu vein with only one bulla. Other characters are difficult to compare since *T. ventrator* is more poorly preserved. *T. bibori* is generally much larger than *T. levii* sp. nov. and has much stouter antennal segments and T1. While *T. levii* sp. nov. has either none or very weak and short latero-median carina on T1, *T. bibori* shows a prominent latero-median carina that exceed half the length of T1.

**Description:** Body 5.6 mm. Head, antenna, mesosoma and metasoma seem dark brown or black. Legs appear bright, yellow, or orange.

**Head.** Face not separated from clypeus, evenly convex to slightly inflated. Mandibles bidentate, length 1.5–2 × base width, apex width 0.4–0.5 × base width, not twisted. Malar space short, 0.35 × mandible base width, without modifications. Maxillary palps with five segments, labial palps with four segments. Clypeus flat, without transverse division, apical margin appears simple. Labrum concealed. Apical tentorial pits small. Upper face with triangular process. Inner orbit appears parallel in frontal view, weakly concave opposite antennal sockets. Eyes without setae, height 0.88 × head height in lateral view. Ocelli seem normal-sized. Genae smooth. Occipital carina complete. Vertex precipitously declivous behind posterior ocelli, flat to concave. Antennae 4.5 mm, with 23–24 segments; scape length 1.2 × width, laterally 1.1–1.2 × pedicle length; first antennal segment length 4.3 × apical width; other antennal segment lengths about 2.8 × apical width. Frons with high lamella between antennal sockets, almost reaching median ocellus.

**Mesosoma.** Pronotum moderately long, about same length as depth, upper posterior corner overhanging sclerite partially or completely; epomia appears absent. Mesoscutum evenly pubescent, finely punctate, carina along lateral margin seems complete; notaulus short, present only on frontal vertical part, shallow. Scutellum more or less flat, with lateral longitudinal carina absent. Mesopleuron strongly convex antero-dorsally; epicnemical carina extends to subtegular ridge; mesopleural furrow more or less straight with isolated puncture; sternaulus absent, posterior ventral corner with a lobe. Mesosternum appears transverse in front of fore coxa, posterior transverse carina absent in front of mid coxa, but with two small lobes protruding on inner side in front of mid coxa. Metapleuron about as long as wide; submetapleural carina complete, anterior section modified into broad lobe; pleural carina present as distinct carina on whole length; juxtacoxal carina absent. Propodeum rounded laterally, about as long as wide; smooth or with weak punctures; hind margin simple; lateromedian and lateral longitudinal carinae present; anterior transverse carina absent, without modifications at junction of lateral longitudinal and posterior transverse carina; posterior transverse carina complete; spiracle separated from pleural carina by less than minimum diameter.

Fore legs ventrally unspecialized; tibia without tooth; 4th tarsomere about as wide as long. Fore and mid trochantellus appear fused with trochanters. Mid tibia with two spurs, inner spur longer than outer. Hind coxa slightly longer than deep; femur 2.4 × longer than wide, without modification; two tibial spurs, inner slender and about 3 × as long as outer; 1st tarsomere 5.6 × longer than apically wide. Claws appear simple; orbicula seems slender, more than 3.5 × as long as wide.

**Wings.** Fore wing 3.9 mm, stalked areolet, closed, quadratic shape, with 4M very short and 3rs-m similar length as 2 + 3M; 2m-cu straight with one bulla anteriorly, which covers 40% of 2m-cu; 4Cu and 5Cu equal in length; 4Rs distally arched; 1m-cu&2Rs + M vein evenly arched, with ramulus absent; 1cu-a strongly postfurcal to 1M + 1Rs with 1Cu 3.5 × 2Cu width, angle to vein 2Cu clearly smaller than 65–70°; pterostigma 2.7 × longer than wide; 2R1 3 × longer as wide; 5M tubular through the whole length; 2Cu 0.64 × 1M + 1Rs, 1.16 × r-rs; 3Cu longer than 2cu-a. Hind wing with M + CU curved around middle or curved along entire length; 1Cu slightly longer than cu-a; 2Rs and 2Cu veins partly spectral; vein 2M completely spectral; 1Rs about 1.6 × rs-m; eight distal hamuli.

**Metasoma.** 3.2 mm, depressed. T1 slightly tapering anteriorly in dorsal view, 1.5–1.6 × as long as posteriorly wide, shape laterally evenly but weakly rounded throughout whole length, but narrower anteriorly; spiracle in anterior half, dorso-lateral carina more or less complete, above or at spiracle; glymma present, rather shallow; laterotergite 1 absent. S1 short, about 0.3–0.4 × length of T1. T1 and T2 separated by normal joint. T2 without latero-median carina, sculpture either smooth, evenly shagreened, or finely punctured; laterotergite creased, about 0.2 × as long as wide. T2–T5 similar in size and dimension, about as long as posteriorly wide. T3 with laterotergite creased, 0.43 × as wide as long. T7 seems flat and evenly sclerotized, 0.67 × length of T6. Parameres 2 × as long as wide at mid length.

Subfamily **Pimplinae** Wesmael, 1845

Genus ***Firkantus*** Viertler, Klopfstein & Spasojevic gen. nov. (masculine)

urn:lsid:zoobank.org:act:93A7E291-93A0-4278-B40B-51F5092551FD

**Etymology:** *Firkantus* is derived from the Danish word “firkant”, meaning rectangle, which indicates the rectangular and square shapes of the areas enclosed by the propodeal carinae.

**Type species:** *Firkantus freddykruegeri* gen. et sp. nov.

**Systematic placement**: The general habitus of this specimen resembles Metopiinae, with a slightly convex face, swollen legs, broad first tergite with spiracle around the middle, and broad and short tergites. The clear division of the clypeus from the face, lack of a triangular process on the dorsal part of the face, as well as no visible ridge between the antennal sockets to median ocellus, and complete propodeal carination (all only detectable after the micro-CT scan) precludes a placement in Metopiinae.

With the complete carination on the propodeum, revealed with the micro-CT scanning, the fossil specimen resembles some taxa in the tribe Tryphonini (Tryphoninae), which sometimes also exhibit a rather stout appearance. Some characters of the fossil, such as the transverse impressed clypeus, stout T1, and the tergites with a broad transverse groove, are found in *Ctenochira* Förster, 1855. What speaks against the placement in *Ctenochira*, or the tribe Tryphonini in general, are the tapered mandibles, the strongly impressed notauli, the simple tarsal claws of the fossil, vein 1Cu being shorter than cu-a (nervellus intercepted above the middle) on the hind wing and the dorsoventrally depressed aedeagus. The very broad laterotergites do appear in some Tryphonini taxa, but this is rather rare.

The fossil also resembles Pimplinae, in the short and deep pronotum, the wing venation, broad laterotergites, dorsoventrally compressed aedeagus and the overall habitus shape. The complete carination can also be found in a few pimpline genera, *Theronia* Holmgren, 1859, *Xanthopimpla* and the extinct genus *Crusopimpla* Kopylov, Spasojevic & Klopfstein 2018. *Firkantus freddykruegeri* gen. et sp. nov. is placed in Pimplinae, which is also in agreement with the phylogenetic placement.

**Diagnosis**: A placement in the extinct genus *Crusopimpla* can be excluded because the new fossil genus has the occipital carina present ventrally, its pterostigma not as broad, and 1Cu shorter than cu-a in the hind wing. Also, *Crusopimpla* species show a rather short 2Cu cell in the fore wing, while our fossil has it 2.6 × as long as wide.

The new fossil genus shares many characteristics with *Lissopimpla* and *Xanthopimpla,* such as the strongly tapered mandibles (Character #1), the deep notauli (Character #57), the presence of lateromedian, lateral longitudinal (Character #76) and posterior transverse propodeal carinae (Character #79), the slender and bar-like orbiculae on the hind claws (Character #124) and the broad laterotergites on T2–T4 (Characters #188, #194 and #198). *Xanthopimpla* and many *Theronia* species possess a yellow-coloured body, with usually black spots or stripes on the metasoma. Unfortunately, in our fossil we cannot determine the ground plan coloration or color patterns. *Xanthopimpla* additionally shares the following characteristics with the fossil specimen: the conspicuously thickened hind femur (Character #110), large tarsal claws (not coded) and nervellus intercepted above the middle (Character #151).

However, our fossil specimen has complete propodeal carination, which is not the case in *Lissopimpla*, *Theronia* or *Xanthopimpla*, which almost always lack the anterior transverse carina, or at least the median portion. *Theronia* can further be excluded because of their broad mandibles, that are not as strongly tapered, and the short, indistinct, or rather shallow notauli. Other characters that are very typical for *Xanthopimpla* and *Lissopimpla*, but are absent in our fossil, are the crest in front of the notauli, eyes with an invagination opposite the antennal sockets in dorsal view, an impunctate and smooth sculpture on T1, and T3 with a transverse, lozenge shaped area, bordered by deep grooves, at least posteriorly.

Because of the previously mentioned morphological disagreements with similar genera, which are also reflected in the phylogenetic analysis, we confidently describe a new pimpline genus *Firkantus* gen. nov.

**Description:** The new genus *Firkantus* is characterized by the evenly convex face separated from the clypeus, the clypeus small and the apical margin without tubercles, denticles or prominent protrusions, the strongly tapered mandibles, the malar space shorter than the mandible base width and the occipital carina ventrally present. The mesosoma has deep converging notauli, reaching the middle of the mesoscutum, the propleuron without a lobe, the epicnemical carina not reaching the anterior margin of the mesopleuron, complete propodeal carination, posterior transverse carina of the mesosternum present laterally and between the mid coxae, metapleuron with the juxtacoxal carina present. The new genus has the hind femur enlarged, large claws, with slender and bar-like orbiculae. The hind wing has 1Cu shorter than cu-a, and the T1 is finely punctate and slightly longer than wide, with the glymma present, the laterotergite triangular and membranous with parallel latero-median carina reaching the posterior end, as well as T2–T4 creased, with lateromedian round swellings and subapical impressions, and the parameres strongly enlarged and longer than most tergites laterally.

**Fig. 7:**
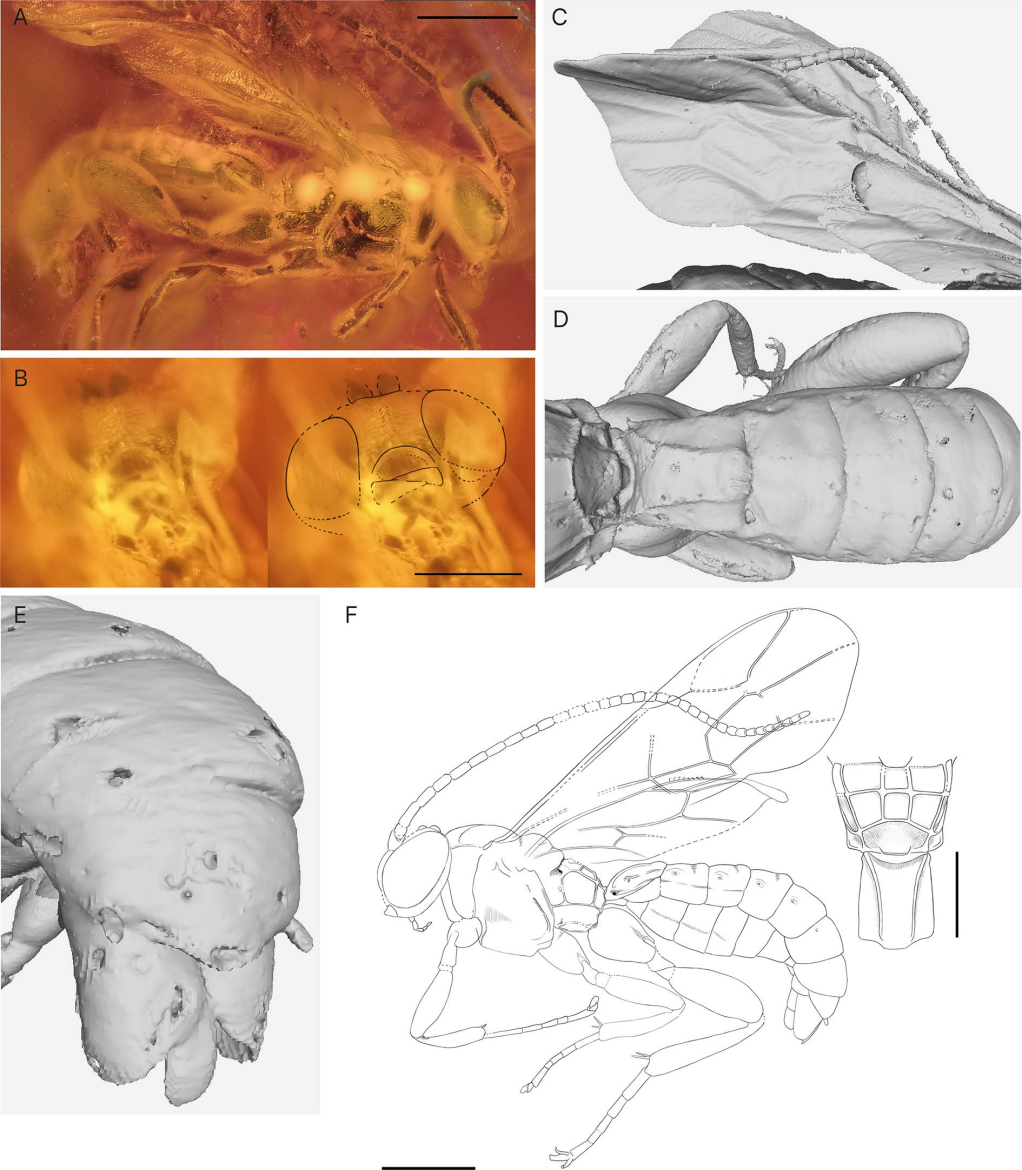
Holotype of *Firkantus freddykruegeri* gen. et sp. nov. **A** Habitus of specimen, lateral view.** B** Anterior view of face, right side with facial structures indicated. **C** Fore wing with folds indicating wing venation. **D** Anterior part of metasoma, dorsal view. **E** Posterior part of metasoma, with parameters and aedeagus. **F** Interpretative drawing with an additional drawing of the propodeum and T1 in dorsal view, where photos and micro-CT scan were used as templates. Scale bar A: 1 mm, B: 0.5 mm, F: lower 1 mm, right 0.5 mm

***Firkantus freddykruegeri*** Viertler, Klopfstein & Spasojevic sp. nov. (Fig. 7)

urn:lsid:zoobank.org:act:3BA05039-B298-4D6D-937F-129D22B1EB10

**Etymology**: Named after the fictional character Freddy Krueger from the horror movie “A nightmare on Elm Street”. Alludes to the shared characteristics of the fictional character and the fossil species—long claws and arolium.

**Type specimen**: Holotype: male (#NHMD_876111, A.K. Andersen, 28–3-1968). Baltic amber. Location: West Coast of Jutland (Denmark). Deposited in Denmark, Natural history museum in Copenhagen.

**Type condition:** The surface has cracks. The holotype is only visible through one side of the amber piece, and details like mouth parts, the propodeal spiracle, and spiracles on T2–T4 are veiled by whitish coatings, often found in Baltic amber (Weitschat & Wichard, 1998). The opposite side is obscured by another inclusion of a Tachyporinae (Staphylidinae) and some blurring.The surface of this amber piece cracked further due to an accident and was cast in resin to stabilize it for future studies.

**Description**: Body 5.7 mm. Colouration difficult to interpret.

**Head.** Face separated from clypeus, evenly convex. Mandibles either unidentate or bidentate with very small lower tooth, strongly tapered with apex width about 0.3–0.5 base width, length 2 × base width; visible tooth looks pointed. Malar space short, between 0.2 and 0.5 × of mandible base width. Maxillary palps with five, labial palps with four segments. Clypeus small, slightly convex or truncate, transverse division unclear, apical margin without tubercles, denticles or prominent protrusions. Labrum concealed. Apical tentorial pits small. Eyes with inner orbits parallel to each other, without setae, height 0.87 × head height in lateral view. Ocelli normal sized. Occipital carina visible ventrally. Genae rather narrow, seems smooth. Antennae at least 5.4 mm, with about 33 segments; more or less evenly thick throughout; scape 1.4 × longer than wide, larger than pedicle; first antennal segment length about 2.8–3.6 × apical width; other antennal segment lengths between 1.7 and 1.4 × apical width.

**Mesosoma.** Pronotum moderately long, slightly deeper than long. Propleuron without lobe. Mesoscutum finely or coarsely punctate, appears evenly pubescent; carina along lateral margin complete to anterior end of scutellum; notaulus distinct, extends to half-length of mesoscutum, converging and meeting other notaulus in middle. Mesopleuron convex; epicnemical carina not curving anteriorly, extends to about lower third of pronotum; sternaulus absent; mesopleural furrow slightly angled opposite episternal scrobe. Mesosternum appears transverse in front of fore coxa; posterior transverse carina present at least laterally and between mid coxae. Metapleuron about 1.5–1.7 × as long as deep; submetapleural carina complete, anterior section unmodified; pleural carina present as distinct carina along whole length; juxtacoxal carina present. Propodeum rounded laterally, about as long as wide, hind margin simple; lateromedian and lateral longitudinal carinae present; anterior and posterior transverse carina complete, carina leading from pleural carina to spiracle present; spiracle shape or position unclear, without modifications. Dorsal part of metacoxal cavity appears to be just at or only slightly above lower margin of metasomal foramen magnum.

Fore leg tibia with 4th tarsomere longer than wide; trochantellus differentiated from femur. Mid leg with two spurs on tibia, spurs equal in length; trochantellus differentiated from femur. Hind leg with coxa 1.2 × longer than deep; femur 2.5 × longer than wide, without modification; tibia slightly enlarged, with two tibial spurs, equal in length; 1st tarsomere 3.5 × longer than apically wide. Claws appear large, simple, with arolium enlarged, projecting conspicuously beyond apex of claw.

**Wings.** Fore wing 4.4 mm, with areolet appearing closed, shape unclear; 2m-cu present, shape appears slightly bowed outwards; 4Rs sinusoidal; 1cu-a interstitial to 1M + 1Rs; 1m-cu&2Rs + M vein angled; pterostigma 3.8 × longer than wide, 0.6 × 1R1; cell 2R1 3 × longer as wide; 5M tubular throughout or partly spectral; 2Cu same length as 1M + 1Rs; 1cu-a 0.45×1M+1Rs; 2Cu 2.8 × longer than wide; 3Cu longer than 2cu-a; 4Cu and 5Cu same length; 2A 2.4 × longer than 1cu-a. Hind wing with 1Cu slightly shorter than cu-a; 2Rs, 2M and 2Cu tubular or partly spectral; 1Rs 1.85 × rs-m; eight distal hamuli.

**Metasoma.** 3.1 mm, cylindrical. T1 finely punctate, 1.2 × longer than posteriorly wide, with stout base, in dorsal view parallel sided, in lateral view with anterior small hump; dorso-lateral carina more or less absent, vestige at anterior and posterior ends; spiracle at 0.44 × T1; glymma present; latero-median carina along whole length, slightly converging before becoming parallel after first third; laterotergite triangular along almost entire length and membranous; S1 length 0.44 × T1. T1 and T2 separated by normal joint. T2 transverse, with deep to fine punctures, laterally 0.7 × length of T1; latero-median carina might be present anteriorly, but uncertain; laterotergite creased, broad, 0.46 × as wide as long; spiracle above crease. T2-T4 with latero-median round swellings and subapical transverse impressions. T3 and T4 with laterotergite creased, broad, about 0.5 × as wide as long. T6 and T7 of similar size. Aedeagus appears dorsoventrally depressed. Parameres enlarged, longer than most tergites laterally; about 1.5 × as long as wide at mid length.

**Diagnosis**: See genus diagnosis.

Subfamily **Rhyssinae** Morley, 1913

Genus ***Rhyssa*** Gravenhorst, 1829

**Fig. 8:**
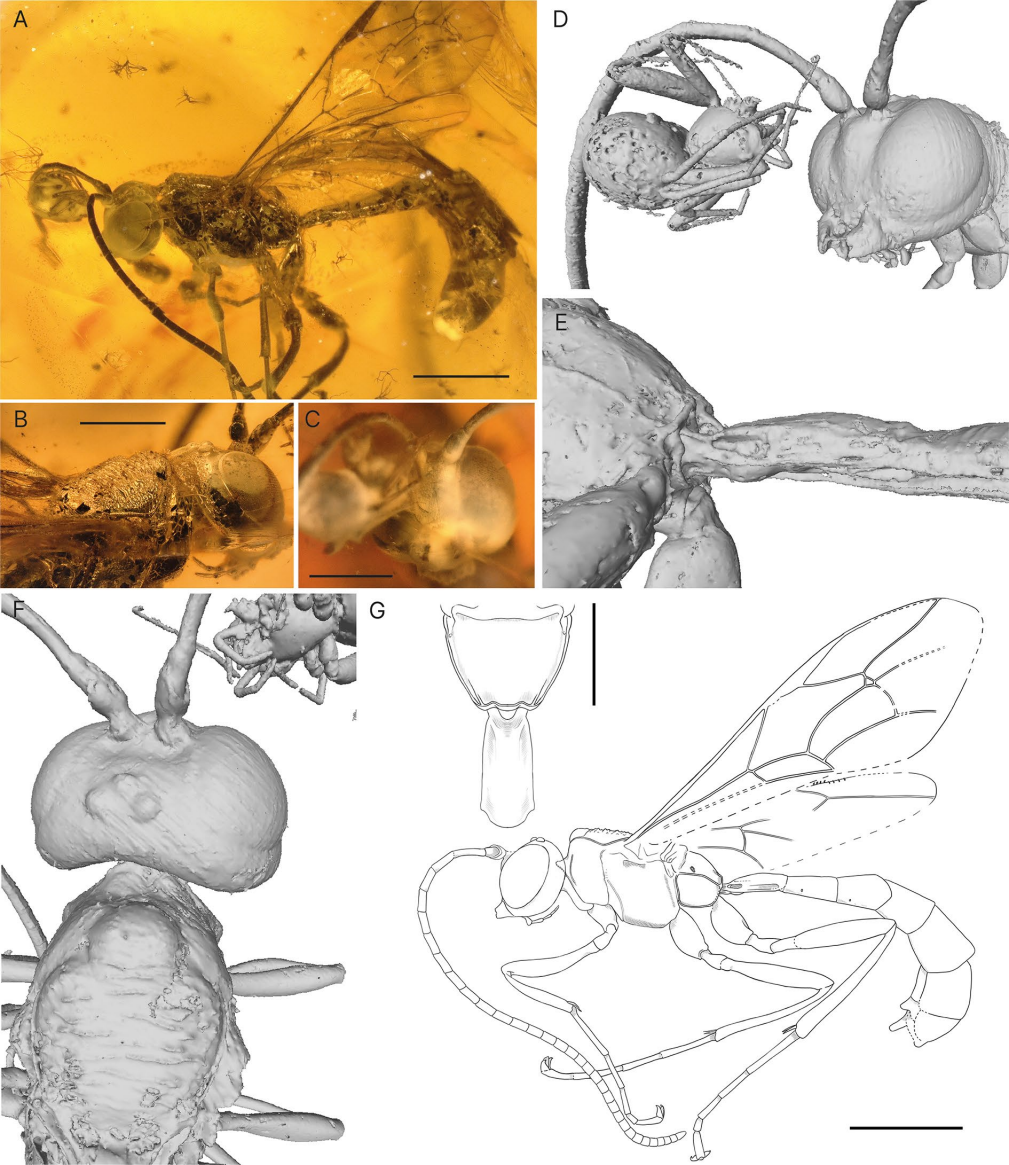
Holotype of *Rhyssa gulliveri* sp. nov. **A** Habitus of specimen, lateral view. **B** Rugae dorsally on mesoscutum. **C** Face, anterior view, partially hidden by spider inclusion and milky coatings. **D** Face, more laterally with visible mandibles. **E** First tergite on metasoma, lateral view.** F** Head and mesoscutum, dorsal view.** G** Interpretative drawing with an additional drawing of the propodeum and T1 in dorsal view, where photos and micro-CT scan were used as templates. Scale bar A: 2 mm, B and C: 1 mm, D: upper 1 mm, lower 2 mm

***Rhyssa gulliveri*** Viertler, Klopfstein & Spasojevic sp. nov. (Fig. 8)

urn:lsid:zoobank.org:act:FB7705BB-0A5E-424A-906E-94B76CFBFE40

**Etymology**: Named after Lemuel Gulliver from Gulliver’s Travels, who was trapped despite his body size.

**Type specimen**: Holotype: male (NMB F3742). Baltic amber. Location: Kaliningrad region (Yantarny, Russia). Deposited in Switzerland, Natural History Museum in Basel.

**Type condition**: Well preserved, with only minor milky coatings on the clypeus. Additional spider inclusion and trichomes, probably from oaks.

**Systematic placement**: The specimen is clearly placed in Rhyssinae due to the distinct transverse rugae on the mesoscutum, dorsally absent occipital carina and the very short 1Cu vein in the hind wing. The chisel-shaped upper tooth (Character #2), the junction of occipital and oral carinae distant from the mandibular base (not coded), the very slender and elongate pterostigma (Character #140), and the presence of a narrow glymma (Character #162), suggest a placement in the genus *Rhyssa* Gravenhorst, 1829. Also, the number of flagellomeres (Character #40), the shape of the anterior part of the pronotum with a longitudinal impression forming a channel (Character #47), and the position of the metacoxal cavity (Character #89) agree with *Rhyssa*. The combined-evidence analysis confirms the placement of the fossil in the extant genus *Rhyssa* with an attachment frequency of over 97% in the RoguePlot.

**Diagnosis**: There are three other fossils described in the genus *Rhyssa*, all of them compression fossils in sediments, and more poorly preserved. *Rhyssa antiqua* Heer, 1867 differs from *Rhyssa gulliveri* sp. nov. by having a very large and non-petiolate areolet, a straight 2m-cu and veins 4Cu and 5Cu of similar length. *Rhyssa*? *juvenis* Scudder, 1890 is preserved poorly and, according to a recent revision (Spasojevic et al. 2018), its body parts seem to belong to several insect specimens. In *Rhyssa*? *juvenis* the only characteristics matching *Rhyssa* are the partially visible fore wing venation and the debatable presence of rugae on the mesoscutum. So, comparing its wing venation with *Rhyssa gulliveri* sp. nov. we find that *R*.? *juvenis* has a more elongate and non-petiolate areolet. *Rhyssa petiolata* Brues, 1906 is described from a female specimen, which has T6 and T7 small and triangular, whereas *Rhyssa gulliveri* sp. nov. is a male and has T6 and T7 similar in size as T4 and T6. Although the wings of *Rhyssa petiolata* are poorly preserved, the areolet appears to be open, in contrast to *Rhyssa gulliveri* sp. nov. and all described extant *Rhyssa* species (exceptions in some small males (Townes, 1969a)).

**Description**: Body 12 mm. Colouration appears dark, either brown or black.

**Head.** Face separated from clypeus, evenly convex. Mandibles bidentate, not twisted, apex width 0.58 × base width, about 1.5 × longer as base width; teeth appear chisel shaped, upper tooth length seems subequal to lower, width of upper tooth equal or slightly broader than lower. Malar space short, 0.3 × of mandible base width. Maxillary palps with five, labial palps with four segments. Clypeus flat, small, without transverse division, apical margin unclear. Labrum concealed. Apical tentorial pits normal sized. Inner orbits parallel in front view, straight opposite antennal sockets. Eyes without setae, height 0.86 × head height in lateral view. Ocelli normal sized. Genae smooth. Occipital carina present ventrally, appears absent dorsally; junction to hypostomal carina distant from mandibular base by about 0.6 × basal mandible. Vertex evenly rounded down to occipital carina. Antennae 9.3 mm, with 32 segments; more or less even thickness throughout; scape from front view truncated at 40° to longitudinal axis, 1.5 × longer than wide (measured at longest/widest point), clearly larger than pedicle; first antennal segment length 4.2 × apical width; other antennal segment lengths about 1.2–2 × apical width.

**Mesosoma.** Pronotum rather short, about 0.68 × longer than deep; epomia appears complete; anterior part rather long, triangularly protruding laterally; longitudinally impressed, forming a channel. Mesoscutum with sharp transverse rugae, appears evenly pubescent; notauli short, not reaching mid length of mesoscutum, strongly converging and deeply impressed anteriorly. Scutellum more or less flat, lateral longitudinal carina seems absent. Mesopleuron flat; epicnemical carina curving anterior but seems not to reach anterior margin of mesopleuron; sternaulus absent. Mesopleural furrow more or less straight with a shallow horizontal impression. Mesosternum appears transverse in front of fore coxa, posterior transverse carina of mesosternum absent. Metapleuron 1.4 × longer than deep; submetapleural carina complete, anterior section unmodified; pleural carina present as distinct carina on whole length; juxtacoxal carina absent. Propodeum dorsally flattened, about as long as wide, hind margin simple; lateromedian carina absent; traces of lateral longitudinal carina posteriorly; anterior transverse carina absent; posterior transverse carina absent; spiracle oval, about 2.2 × longer than wide, separated from pleural carina by about its minimum diameter. Metacoxal cavity with dorsal margin above lower margin of metasomal foramen magnum.

Fore legs ventrally unspecialized; tibia without tooth; 4th tarsomere longer than wide. Fore and mid tibia with scattered long and stronger spines. Mid tibia with row of strong spikes on inner side apically; with two spurs, equal in length. Hind coxa 1.76 × longer as wide; femur 4.75–5.2 × longer than wide, without modification; tibia with two spurs, inner slightly shorter than outer; 1st tarsomere 10.5 × longer than apically wide. Claws appear simple.

**Wings.** Fore wing 8.7 mm. Areolet closed, strongly petiolate, oblique quadratic shape with 4M very short and 3rs-m similar length as 2Rs; 2m-cu slightly and evenly bowed outwards, with two bullae, that cover 20% of 2m-cu and are evenly distributed; vein 3rs-m with one bulla; 4Cu 1.9 × 5Cu; 4Rs appears sinusoidal; ramulus appears absent; 1cu-a postfurcal to 1M+1Rs with 2Cu 2 × 2Cu width, angle to vein 2Cu clearly smaller than 65°–70°; pterostigma 6.8 × longer than wide, 0.8 × 1R1; cell 2R1 4.2 × longer than wide; 5M tubular; vein 2Cu 0.92 × 1M + 1Rs, 0.94 × r-rs; cell 2Cu 4.4 × longer than wide; 1m-cu&2Rs+M vein arched; 3Cu similar length than 2cu-a. Hind wing with cu-a 5.3 × 1Cu; veins 2Rs, 2M and 2Cu appear tubular throughout; 1Rs 1.6 × rs-m.

**Metasoma.** 7.7 mm, depressed to cylindrical. T1 parallel-sided in dorsal view, 1.8 × as long as posteriorly wide, evenly but weakly rounded throughout whole length in lateral view; glymma present; latero-median carina absent. S1 about 0.5 × T1. T1 slightly longer than T2 dorsally, separated by normal joint. T2 1.1 × as long as posteriorly wide; latero-median carina absent; laterotergites creased but appear more or less absent. T3–T6 subquadrate in dorsal view. T6 and T7 similar in size. S7 flat and evenly sclerotized. Apex of aedeagus appears dorsoventrally depressed.

Subfamily **Phygadeuontinae?** Förster, 1869 (sensu Santos (2017))

Genus ***Magnocula*** Viertler, Klopfstein & Spasojevic gen. nov. (feminine)

urn:lsid:zoobank.org:act:D2B1B906-279E-440E-B666-F5984022A986

**Etymology**: The name is a combination of the latin words “magna” and “ocula”, meaning “large-eyed”.

**Type species**: *Magnocula sarcophaga* gen. et sp. nov.

**Systematic placement:** The long and narrow T1, with its sternite fused and without glymma resembles some Diacritinae. Also, the fossils’ strong notaulus and the small areolet are characteristics found in Diacritinae. However, the taxa in this subfamily do not possess a complete sternaulus, are at least double the size, and have their nervellus intercepted, which is not the case in our fossil. The fossil also resembles *Adelphion* Townes, 1969 in Pedunculinae, with a similarly looking clypeus, wing venation, and T1. But the notaulus shape disagrees with this genus, as well as the dimension of the laterotergite 2 and 3, and the shape of the ovipositor tip.

The fossil does also resemble some Orthocentrinae, with its setae on the ovipositor sheaths about as long as the sheath width or slightly shorter. Specifically, the short carina anterior of the notauli, and the shapes of the hypopygium and the ovipositor are similar to *Symplecis* Förster, 1869. But despite the similarities, the fossils mandibles do not agree with Orthocentrinae. Unfortunately, the dense fringe on the apex of the hind tibia, a character that would further support the placement in Orthocentrinae, is not discernible.

The fossil resembles taxa in the extinct subfamily Townesitinae, which is only known from Baltic amber. The similarities include the extremely small body size, tapered mandible, narrow temples, seemingly elongate scapus, long sternaulus, fully areolated propodeum, and the slender legs. However, the strongly developed epomia, together with the wing venation does not fit Townesitinae, which normally possess a very short r-rs vein, and generally stout and short 1 M + 1R1 and 2 M cells.

A long sternaulus, which reaches back to the posterior end of mesopleuron, is not only found in Townesitinae, but also in two extant subfamilies, Cryptinae and Phygadeuontinae. The sternaulus, which ends posteriorly above the mid coxa and the two bullae in vein 2m-cu, means the fossil resembles Phygadeuontinae more than Cryptinae. The quadratic and slightly petiolate areolet is still quite rare in both Phygadeuontinae and Cryptinae, and the observed shape of the first tergite, with the spiracle around the middle, is also not common in this group and points to a potentially ancestral position. Ancestral Phygadeuontinae seem to exhibit more plesiomorphic character combinations, as was already observed in the extinct genus *Madma* Viertler, Klopfstein & Spasojevic, 2022 (Viertler et al., 2022b).

The fossil combines characteristics of many extant genera and tribes of Phygadeuontinae, which are in agreement with the phylogenetic placement. However, given the similarities to some Orthocentrinae and Pedunculinae, and lack of some subfamily-specific morphological characters due to taphonomic processes, we add a question mark behind the subfamily name.

**Diagnosis:** Within Phygadeuontinae, the fossil seems to combine characteristics from different extant groups. With the rather deep and strongly converging notauli (Character #57 and # 58), the elongate area superomedia (not coded) and the very long first sternite (Character #174), the fossil resembles some genera previously placed in Chiroticina. But species of Chiroticina have mostly elongated maxillary palps and a long malar space, which is not the case in our fossil.

Phygadeuontine genera previously placed in the tribe Hemitelina can be excluded since they have the spiracle behind the middle and have their laterotergites 2 and 3 not creased.

The slender T1 with the spiracle around the middle (Character #164 and #171) fits some genera previously placed in Bathytrichina. Many share characteristics with the fossil, such as a rather wide head (not coded) and an elongate area superomedia (not coded). Those genera have their pedicel rather large compared to the scape, but we are uncertain in the interpretation of pedicel and scape in our fossil, which might be either normal in dimensions or enlarged. We can exclude *Apophysius* Cushman, 1922 due to the single bulla in 2m-cu; *Bathythrix* Förster, 1869 due to weakly converging notauli, that reach only the centre of the mesoscutum; *Chrysocryptus* Cameron, 1902 due to the teeth length and the clypeus with modifications; *Retalia* Seyrig, 1952 and *Rhabdosis* Townes, 1970 due to not having propodeal apophyses; and *Surculus* Townes, 1970 due to the absence of the basal section of the lateral longitudinal carina and no propodeal apophyses. In addition, the quadratic and slightly petiolate areolet (Character #129) is not found in any taxa of Bathytrichina, or any other extant tribe. We therefore suggest a new extinct genus, *Magnocula* gen. nov.

*Magnocula gen. nov.* differs from the only other described extinct Phygadeuontinae, genus *Madma* Viertler, Klopfstein, & Spasojevic 2022, by not having a bilobed posterior transverse carina on the mesosternum, no tooth on the apical margin of the fore tibia and by having a quadratic areolet. *Magnocula* gen. nov. shows less plesiomorphic characteristics than *Madma*, thus, in contrast to *Madma*, we suggest it is a crown than stem Phygadeuontinae. In conclusion, since this fossils characteristic combination is not found elsewhere and the fossil shows quite a special quadratic areolet shape for the subfamily, we suggest a new genus.

**Description:** The new genus *Magnocula* gen. nov. exhibits a combination the following: Bidentate mandibles and the malar space 0.7 × of the mandible base width, the clypeus slightly convex without modifications on the apical margin, the eyes are large with 0.8 × head height in lateral view, the occipital carina complete and antenna with 15–17 segments. The notaulus is deep and strongly converging to the middle of the mesoscutum. The epomia is rather strong in *Magnocula* gen. nov. and ventrally on a slight elevation. Sternaulus present and ending above the mid coxa, the posterior transverse carina of the mesosternum absent in front of mid coxa but present otherwise. Juxtacoxal carina present, as well as the lateromedian, lateral longitudinal and both transverse carinae. The fore wing with quadratic areolet, which is slightly petiolate, 2m-cu bowed outwards with two bullae, and 1cu-a meets 1 M + 1Rs interstitial. 2Cu absent in the hind wing. S1 almost reaching the posterior part of T1, T1 slender and with the spiracle around the middle. T2 with gastrocoelus. Laterotergites of T2–T5 creased.T8 is elongate dorsally, not forming a horn or boss. The length of the ovipositor sheath is around 0.3 × the metasoma length.

**Fig. 9:**
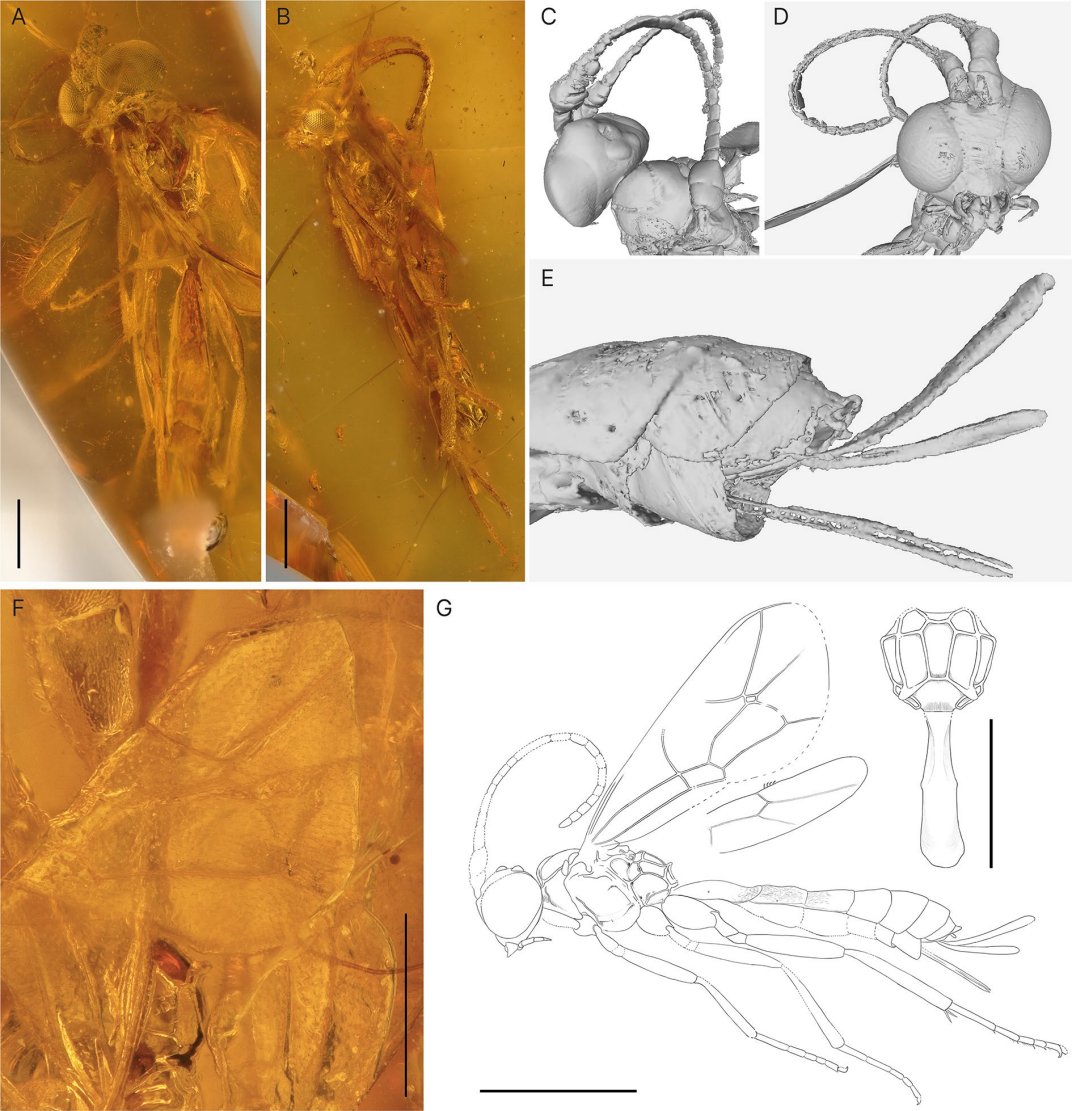
Holotype *Magnocula sarcophaga* gen. et sp. nov. **A** Habitus of specimen, ventral view. **B** Habitus of holotype, lateral view. CT scan of **C** head and mesoscutum in dorsal view, **D** face in anterior view, and **E** last tergites with ovipositor and sheaths. **F** Photo of a partial fore wing, in top left is T2 with its rugopunctate to striate structure. **G** Interpretative drawing with an additional drawing of the propodeum and T1 in dorsal view, where photos and micro-CT scan were used as templates. Scale bar A: 1 mm, F: 0.5 mm G: lower 1 mm, right 0.5 mm

***Magnocula sarcophaga*** Viertler, Klopfstein & Spasojevic sp. nov. (Fig. 9)

urn:lsid:zoobank.org:pub:2F370DF6-6B5C-4B69-A670-51BDD54414A4

**Etymology**: Derived from the coffin-shaped area superomedia.

**Type specimen**: Holotype: female (#NHMD_876130, A.K. Andersen, 28–3-1968). Baltic amber. Location: West Coast of Jutland (Denmark). Deposited in Denmark, Natural history museum in Copenhagen.

**Type condition**: Specimen complete, bubbles covering some parts anteriorly. Head, mesoscutum partially oblique and distorted due to preservation. Wings folded partially and covering themselves in dorsal and lateral view. The amber piece fell apart in three pieces due to an accident and all pieces were cast separately in epoxy to stabilize them for future studies and observations. Because this amber fossil was mummified, the accident revealed body parts that were not visible before the micro-CT scanning. The additional pictures of the damaged holotype can be found in the Additional file 11.

**Description:** Body 3.2 mm. Colouration difficult to interpret.

**Head.** Face separated from clypeus, evenly convex. Mandibles bidentate, not twisted, teeth simple, pointed, upper tooth equal in length and width to lower. Malar space 0.7 × of mandible base width. Maxillary palps with five segments. Clypeus slightly convex, wider than long, without transverse division, apical margin simple. Labrum concealed. Apical tentorial pits small to normal-sized. Inner orbits converging in front view, straight opposite antennal sockets. Eyes without setae, height 0.8 × head height in lateral view. Genae smooth. Occipital carina seems complete; junction to hypostomal carina distant from mandibular base by 1.2 × basal width of mandible. Antennae 2.3 mm, with 15–17 segments; appears more or less even thick throughout; scape and pedicel concealed; first two antennal segments more than 3 times as long as wide; other antennal segment lengths about 1.2–1.8 × apical width.

**Mesosoma.** Pronotum rather short and deep, about 0.6 × longer than deep; epomia complete and prominent, reaches horizontal part of pronotum. Mesoscutum finely punctate, evenly pubescent, with carina along lateral margin complete to anterior end of scutellum, with a short, parallel carina directly anterior to base of notaulus; notaulus deep, strongly converging, joining other notaulus, extending to about middle of mesoscutum. Scutellum flat to slightly convex, with lateral longitudinal carina absent. Mesopleuron rather flat; epicnemical carina vertical to around mid-height of pronotum; mesopleural furrow more-or-less straight, with a shallow horizontal impression extending from this angulation to scrobe; sternaulus present, ending above level of mid coxa. Mesosternum transverse in front of fore coxa, posterior transverse carina of mesosternum absent in front of mid coxae, prominent between mid coxae. Metapleuron about as long as deep; submetapleural carina complete, anterior section broadened triangular; pleural carina present as distinct carina along whole length; juxtacoxal carina present as distinct carina with additional smaller carinulae. Propodeum rounded laterally, about as long as wide, sculpture smooth with some weak punctures where setae are inserted, lateromedian and lateral longitudinal carinae present, anterior transverse carina complete but shallow, posterior transverse carina complete; area superomedia coffin-shaped; spiracle subcircular, separated from pleural carina by about inner diameter of spiracle, additional carina connecting spiracle with pleural carina and lateral longitudinal carina. Junction of lateral longitudinal and posterior transverse carinae modified as apophysis. Metacoxal cavity with dorsal margin dorsal to ventral margin of metasomal foramen magnum.

Fore legs ventrally unspecialized; tibia without tooth; 4th tarsomere longer than wide. Mid legs with two spurs on tibia, equal in length. Hind leg with coxa 1.9 × longer as wide; femur about 5 × longer than wide, without modification; two tibial spurs, equal in length; 1st tarsomere btw. 7.6–8.8 × longer than apically wide. Claws appear simple.

**Wings.** Fore wing 2.2 mm, areolet closed, quadratic, short petiolate, with 3rs-m and 2+3M similar in length and double the length of 2Rs and 4M; 2m-cu slightly bowed outwards, with two bullae, evenly distributed, that cover together 20% of 2m-cu; 4Cu 2.2 × 5Cu; 4Rs almost straight, without ramulus; 1cu-a meeting 1M + 1Rs interstitial; cell 2R1 2.1 × longer than wide; 5M tubular throughout; 1M+ 1Rs arched; 3Cu longer than 2cu-a. Hind wing with 2Cu absent; 1Rs 0.87 × rs-m; one basal hamulus; four distal hamuli.

**Metasoma.** 1.9 mm, depressed. T1 petiolate to evenly tapering to anterior in dorsal view, elongate, 3.5 × as long as posteriorly wide, posteriorly with rugose punctate to striate sculpture, laterally rounded throughout and narrower anteriorly than in posterior half; glymma absent; latero-median carina absent; spiracle at around mid-length. S1 length 0.9 × T1; seems fused to T1 without visible suture. T1 length 1.6 × T2 dorsally, separated by normal joint. T2 1.2 × as long as posteriorly wide; rugose punctate to striate dorsally; gastrocoelus present, thyridium possibly present and large; latero-median carina absent; laterotergite creased and folded under, about 0.27 × as wide as long. T3 rugose punctate to striate. Laterotergites of T3–T5 also creased and folded under, moderately broad between 0.25 and 0.35 × as wide as long. T4–T6 finely punctured, punctures separated by at least 4 × their diameter. T6 shorter or of similar size to T7. T8 elongate dorsally. Hypopygium appears transverse or about same width as length ventrally. Ovipositor sheaths 0.3 × metasoma length; with dense pubescence and setae about as wide as sheath or slightly shorter; more-or less parallel sided. Ovipositor parallel sided, tip more or less straight; apex modifications uncertain, but without nodus.

**Diagnosis**: See genus diagnosis.

## Discussion

### Comparison before and after micro-CT scanning

In our analysis, the additional morphological information did not have a great impact on the phylogenetic placement of our amber fossils, which all retained the original subfamily placement. The heterogeneity of the improvement in the precision of the placement could be explained by different degrees of preservation quality of our fossils. However, overall, the fossils might have been rather too well preserved to realise the full potential of micro-CT scanning, as the visible characters were already sufficient for an accurate placement. Interestingly, before the micro-CT scanning, most of the inclusions were visible laterally from at least one angle, suggesting that the hidden body aspects, which were mostly dorsal and ventral, were not as significant for improving the phylogenetic placement as we initially thought, at least at a subfamily level. Several studies on other organismic groups did show that micro-CT scanning can reveal significant characteristics which helped in the taxonomic placement of fossil species (Garwood et al., 2017; Henderickx et al., 2013; Moser et al., 2021), but to our knowledge, none of them compared the phylogenetic placement before and after the scanning.

So when could we profit the most from micro-CT scanning to confidently place fossil species? Assuming the fossil is well-enough preserved, the scans seem not always necessary for classification at higher taxonomic levels, and a researcher must carefully weigh potential benefits and costs of this technique. In cases where the fossils are not as easily observable through a light microscope, micro-CT scans are of great importance to study fossil specimens (Dierick et al., 2007). This applies even more for different groups of organisms in which diagnostic characteristics are often hidden, for examples in beetles, for which internal structures are needed for identification (Alekseev & Bukejs, 2021; Kundrata et al., 2020). Furthermore, the usefulness of micro-CT scanning is not only restricted to amber fossils. For example, it can be used to reveal new species in completely opaque structures, such as in coprolites (Qvarnström et al., 2021), or discover additional morphological information from compressed sediment fossils that are preserved in 3D to some extent (Garwood et al., 2016). Furthermore, a 3D reconstruction can be explored from various orientations, and, when publicly available, it offers access for other research questions that are not covered in the original publication. On the downside, micro-CT scanning requires access to a specialised facility and expertise (Racicot, 2017), and depending on the object and the status of the preservation, it can be quite time-consuming to reconstruct the 3D model of a specimen.

Overall, micro-CT scans offer a great opportunity to get detailed access to morphological characteristics to classify and/or or to describe fossil species, as well as to re-describe poorly described amber species. So, if time, costs, and accessibility allow, we highly recommend conducting micro-CT scans for fossil specimens when important morphological characters are not accessible through classical optical microscopes, since they do greatly increase the amount of morphological information.

### Diversity of Darwin wasps in amber

Three of the four newly described fossil species are especially interesting to science since they represent the first species records of their subfamilies in their respective amber locality, while also preserving a high degree of morphological detail. With *Triclistus levii* sp. nov., we describe the first fossil Metopiinae in amber, and the first clear ichneumonid in Dominican amber. Both character evidence and phylogenetic analysis strongly support the placement of this fossil in the extant genus *Triclistus*, which fits the observations that many organisms found in Dominican amber appear morphologically similar to extant groups at lower taxonomic levels (Iturralde-Vinent, 2001).

Since Baltic amber is the largest Cenozoic repository of fossil insects, it is not surprising that ichneumonid fossils are abundant in this amber (Kasparyan & Humala, 1995; Kasparyan, 1988, 1994; Manukyan, 2019, 2023; Manukyan & Zhindarev, 2021). However, just a small portion of the specimens has been described, belonging to only seven (Hybrizontinae, Orthocentrinae, Pherombinae, Pimplinae, Stilbopinae, Townesitinae, and Tryphoninae) of the 47 subfamilies of Ichneumonidae, with many more expected (Manukyan & Zhindarev, 2021). Regarding Dominican amber, there was only one questionable ichneumonoid species described so far, namely *Masona pyriceps* (van Achterberg, 2001). But a recent study recovered this fossil species clearly within Braconidae (Jasso-Martínez et al., 2022). As for Baltic amber, we here add two additional Darwin wasp subfamilies to the seven previously recorded (Manukyan, 2023; Manukyan & Zhindarev, 2021), namely Phygadeuontinae and Rhyssinae. *Magnocula sarcophaga* gen. et sp. nov. is the second ever described Phygadeuontinae in amber, after *Madma oisella* from Oise amber (Viertler et al., 2022b). In Baltic amber, Phygadeuontinae are among if not of the most frequently found subfamily (Manukyan & Zhindarev, 2021), but none of the species have to date been described. This is probably because of their huge extant diversity (Broad et al., 2018; Santos, 2017), which makes proper classification difficult. We hope that our result here will motivate further studies into this interesting group, which will certainly reveal many more new species and potentially even genera of Phygadeuontinae from amber.

The descriptions of fossils belonging to Rhyssinae and Pimplinae in amber especially are exciting since species from these subfamilies are normally rather large in body size and are thus rarely found in amber (Martínez-Delclòs et al., 2003; but see Manukyan, 2023). And while the subfamily placement of *Rhyssa gulliveri* sp. nov. was clear a priori, this was not the case for *Firkantus freddykruegeri* gen. et sp. nov. since we observed many plesiomorphic characters in this fossil that not only occur in Pimplinae, but also in Metopiinae, Tryphoninae and Ctenopelmatinae. Since the first Pimplinae amber fossil was only described very recently (Manukyan, 2023), we did not consider Pimplinae as the most likely candidate subfamily at first, but were rather leaning towards it belonging to one of the basal ophioniform subfamilies. This highlights the importance of considering different approaches, such as a combined-evidence phylogenetic analysis as done here, or a fore wing morphometric analysis as in Viertler et al. (2022a), for classifying fossil specimens, as these might constitute more objective placement methods.

## Conclusion

The accurate placement of fossils is highly relevant for many phylogenetic analyses, as it ensures the accuracy and validity of evolutionary reconstructions. The question on when it is necessary to conduct micro-CT scanning of amber inclusions to increase certainty of taxonomic placement depends on the organism of interest. In the case of Darwin wasps, it seems usually unnecessary for subfamily placement when the lateral view is available. Nevertheless, even in the rather clearly visible amber inclusions we studied, micro-CT scans were relevant for placement at lower taxonomic levels and to test their relationships to extant genera or species.

Even though in our case phylogenetic placements did not change as much as expected with micro-CT scanning, the scans revealed additional morphological characters which enriched the descriptions of the four fossils. This information might help to reconstruct ancient ecologies and character evolution in future studies, since sometimes a single newly available character can give insights into the ecology of a species. In addition, digital open access to the detailed morphological information of specimens allow the easy use of the species in further studies.

### Supplementary Information

**Additional file 1. **Taxon list, showing all included extant and fossil taxa, the genes which were amplified for each taxon, and the GenBank Accession number for the additionally added sequences.

**Additional file 2. **Descriptions of coded morphological characters.

**Additional file 3. **Taxon and character sampling of the data set.

**Additional file 4. **Morphological matrix.

**Additional file 5. **Molecular sampling of additional taxa, and PCR conditions.

**Additional file 6. **A majority rule consensus tree with all compatible groups added and with all fossils from the combined evidence analysis after the scan.

**Additional file 7. **Script for the combined evidence analysis in MrBayes.

**Additional file 8. **Data matrix used for the combined evidence analysis after the scan, with maximum morphological coding.

**Additional file 9. **Attachment distribution and frequency for the four new fossil species, before the scan.

**Additional file 10. **Attachment distribution and frequency for the four new fossil species, after the scan.

**Additional file 11. **Additional photos of *Magnocula* *sarcophaga *gen. et sp. nov. after destruction. **A** Habitus of broken specimen in dorsal view. **B** Revealed head with eye. **C** Revealed partial metasoma with visible structures on T2-T4. **D** Habitus after epoxy cast to stabilize specimen in dorsal view. Scale bar: A: 1mm, B: 0.5mm, C: 0.5mm, D: 1mm.

## Data Availability

The two studied fossils, *Firkantus freddykruegeri* gen. et sp. nov. and *Magnocula sarcophaga* gen. et sp. nov. are deposited at the Natural History Museum of Denmark (#NHMD_876111 and #NHMD_876130), *Rhyssa gulliveri* sp. nov. at the Natural History Museum Basel (NMB F3742) and *Triclistus levii* sp. nov. at the Staatliches Museum für Naturkunde Stuttgart (#DO-3441-M). All data generated or analyzed during this study are included in this published article and its Additional information files. The 3D models of the four new fossil species described in the current study are available on the MorhoBank Repository (http://morphobank.org/permalink/?P4882).
