# Supplementary material for: Impact of increasing morphological information by micro-CT scanning on the phylogenetic placement of Darwin wasps (Hymenoptera, Ichneumonidae) in amber
Source: Swiss J Palaeontol. 2023 Nov 3;142(1):30. doi: 10.1186/s13358-023-00294-2 (PMC10624732; doi:10.1186/s13358-023-00294-2)
Supplement: Supplementary file 2 — Additional file 2. Descriptions of coded morphological characters. [file 13358_2023_294_MOESM2_ESM.docx]

Description of morphological characters

(**modified** Klopfstein & Spasojevic, 2019) = when new states were added or character rephrased.

(**split** from Klopfstein & Spasojevic, 2019) = when a character was split in two characters.

Newly added character

**Head**

**#1 Mandible, dimension [ordered]: (Klopfstein & Spasojevic, 2019)**(0) moderately large, weakly, and evenly tapered so apex is more than 0.7x base;
(1) moderately large, but more strongly tapered so apex is about 0.6-0.5x base;
(2) slender and very strongly tapered so apex is <0.4 times width at base.

**#2 Mandible, shape:** (Klopfstein & Spasojevic, 2019)
(0) bi-dentate, both tips slighly pointed to evenly rounded (includes twisted mandibles, when second tooth is present on the inside but not visible);
(1) bi-dentate, one of the tips abruptly broken (chiesel-shaped);
(2) unidentate, tooth abrubtly broken (chiesel-shaped);
(3) tridentate (upper tooth subdevided);
(4) unidentate, strongly shortened so that when closed, the mandibles barely overlap or not at all (Fig. XX - Skiapus, also in some species of Orthocentrus)
(5) weakly sclerotized - reduced to a flap (Fig. XX Hybrizon rileyi photo )

**#3 Mandible, teeth, length of upper tooth compared with lower tooth [ordered]:** (Klopfstein & Spasojevic, 2019)
(0) upper tooth conspicuously the longer, the lower tooth small, <0.5 of length of upper tooth, to absent;
(1) upper tooth subequal to the lower (Fig. 27);
(2) upper tooth distinctly shorter than the lower tooth, about 0.6– 0.7 of its length (Fig. 23);
(3) upper tooth very small, <0.4 length of lower.

**#4 Mandible, teeth, width of upper tooth relative to lower tooth [ordered]:** (Klopfstein & Spasojevic, 2019)
(0) upper tooth more slender than the lower tooth;
(1) upper tooth similar to or very slightly broader than the lower;
(2) upper tooth conspicuously broader than the lower tooth (Fig. 24), lower might even be absent;

**#5 Mandible, amount of twist around longitudinal axis [ordered]:** (Klopfstein & Spasojevic, 2019)
(0) not twisted (Fig. 27);
(1) weakly twisted 20–40° (Fig. 26), sometimes lower tooth being shifted somewhat inwards compared to upper;
(2) twisted about 90° so the inner tooth is not visible when mandibles closed (Fig. 32).

**#6 Mandible, surface, dorsal chaetotaxy:** (modified from Klopfstein & Spasojevic, 2019)
(0) with a cluster of stout long setae in a pit (Fig. 25);
(1) with sparse scattered pubescence, sometimes glabrous (Fig. 26);
(2) with a diagonal line of hairs starting where the pit usually is and going down and towards the tip (see Ophion).

**#7 Mandible, apical 0.7 of length, surface sculpture:** (modified from Klopfstein & Spasojevic, 2019)
(0) more or less punctate or slightly rugulose (also due to hair) (Fig. 26);
(1) strongly striate;
(2) all smooth (no hair/setae?) (some Orthocentrinae, Diplazontinae).

**#8 Malar space, modification:** (modified from Klopfstein & Spasojevic, 2019)
(0) without any structure or with an incomplete sculptured band;
(1) with a sculptured band in the place where the sulcus/impression would be, band is complete (extending over entire length);
(2) with a deep subocular sulcus/impression.

**#9 Malar space, length relative to mandible width [ordered]:** (modified from Klopfstein & Spasojevic, 2019)
(0) very short, lower margin of eye almost touching base of mandible, at most 0.2 x base of mandible;
(1) short to moderately long, 0.2– 0.8x basal mandibular width (Gauld ea 2002: Figs 27–29);
(2) long, 1-1.5 x basal mandibular width (Gauld ea 2002 Fig. 45); (3) very long, more than than 1.8x basal mandibular width.

**#10 Maxillary palp, number of segments [ordered]:** (Klopfstein & Spasojevic, 2019)
(0) five;
(1) four;
(2) three.

**#11 Labial palp, number of segments:** (Klopfstein & Spasojevic, 2019)
(0) four;
(1) three.

**#12 Clypeus, in lateral view, shape:** (modified from Klopfstein & Spasojevic, 2019)
(0) from flat (in same plane as lower face) to weakly but evenly convex;
(1) evenly convex to about 3/4, then slight impression with apical protrusion;
(2) strongly convex basally, remainder protruding from face, beak-like (cf. Helictes-group in Orthocentrinae);
(3) convex, thickened all the way (cf. Ophion, Xestopelta);
(4) basally slightly swollen then slightly concave apically, so in median longitudinal section it would be slightly sinuous (Fig. 44*);
(5) basally flat (in same plane as lower face), apically protruding, so clypeus overall looks slightly concave (cf. Heteropelma);
(6) strongly and evenly protruding  from base to middle, apical part  evenly restricted, thus in profile triangular (cf. Xenoschesis);
(7) flat in the same plane as the lower face but at the very apex thickened;
(8) opposite of (5) with apical part flat, basal part slightly convex, so clypeus overall looks slightly concave (cf. Tryphon obtusator).

**#13 Clypeus, shape, in front view:** (modified from Klopfstein & Spasojevic, 2019)
(0) undivided, transverse to lenticular, clearly wider than long;
(1) undivided, small and almost quadrate, nearly as high as wide;
(2) transversely divided, with apical part smaller to equal to basal part, the two parts more or less equally sclerotised (cf. Xanthopimpla, Lissopimpla, Hellwigia);
(3) transversely divided, with apical part larger than basal part, and less? sclerotised then basal part (some Labeninae, some Xoridinae);
(4) transversely divided, two parts equally sclerotised with apical part transverse (Fig. x, in tiny Cretaceous ichneumonid, 3311/856b);
(5) entire, subquadrate, but larger than state 1 (cf. Helictes-group Orthocentrinae);
(6) narrow longitudinal, median part protruding and elongate, lateral parts in plane with face (cf. Hybrizontinae);
(7) entire, very wide and abruptly truncate at apex (cf. most Ichneumoninae).

**#14 Clypeus, apical margin, general form:** (modified from Klopfstein & Spasojevic, 2019)
(0) simple, truncate to slightly convex or slightly concave, without tooth or tubercle;
(1) slightly convex and medially pointed;
(2) with small acute lateromedian denticles;
(3) with central part weakly sclerotized and more or less notched, laterally bilobed;
(4) with a median tubercle while otherwise truncate or with weak lateral lobes;
(5) strongly convex without a lobe (some fossils);
(6) with two lateral tooth-like protrusions (see Heteropelma);
(7) as 0 but apical margin weakly sclerotized;
(8) convex, medially thickened (Astiphromma);
(9) laterally bilobed, central part broadly truncated

**#15 Clypeus, apical margin, number:** (Klopfstein & Spasojevic, 2019)
(0) single margin;
(1) double margin.

**#16 Clypeus, apical margin, vestiture:** (modified from Klopfstein & Spasojevic, 2019)
(0) with small scattered setae or lacking setae;
(1) with regularly spaced strong setae (Fig. XX Hercus);
(2) with extremely strong, spike-like setae.

**#17 Clypeus, labrum, visibility [ordered]:** (Klopfstein & Spasojevic, 2019)
(0) mostly concealed below clypeus, at most narrowly visible, weakly sclerotized;
(1) clearly exposed below clypeus, but not strongly sclerotized;
(2) exposed below clypeus, as strongly sclerotized.

**#18 Face, tentorial pits, size [ordered]:** (Klopfstein & Spasojevic, 2019)
(0) very small, indistinct;
(1) middle size, length between 1/3 and 1/2 of the breadth of the first flagellomere
(2) large, longer then 1/2 of the breadth of the first flagellomere.

**#19 Face** form: (Klopfstein & Spasojevic, 2019)
(0) separated from clypeus, simply convex or flat, with a weak median swelling;
(1) separated from clypeus, biconcave lateromedially, with a central and pair of lateral ridges;
(2) separated from clypeus by groove, and strongly protuding (Strongylopsys?);
(3) not separated from clypeus, flat or with weak median swelling;
(4) not separated from clypeus, strongly inflated, very convex in profile (cf. Hypsicera in Metopiinae); (5) not separated from clypeus, strongly protruding at the level of toruli, the lower part in profile more or less straight or at most slightly convex (cf. Orthocentrus-group);
(6) not separated from clypeus, whole surface protruding like a plate (Schyzopyga);
(7) with very deep emargination dorsally and laterally of clypeus (cf. Dialipsis);
(8) clypeus and lower face together forming a nose-like protrusion (genus Zagryphus of Tryphoninae);
(9) shaped like a shield (genus Metopius).

**#20 Face, front view, dorsal end, process between antennal sockets:** (modified from Klopfstein & Spasojevic, 2019)
(0) absent;
(1) upper face produced into a triangular process that extends between or over the bass of the antennae (cf. most Metopiinae);
(2) forming a little gap in the middle (some Ctenopelmatinae?);
(3) with a small median horn.

**#21 Eyes, dorsal view, inner margins of eye, invagination:** (Klopfstein & Spasojevic, 2019)
(0) straight or weakly concave opposite antennal socket;
(1) with a deep, more or less V-shaped invagination opposite antennal socket.

**#22 Eyes, front view, inner margins, convergence (shortest distance related to largest distance between eyes):** (modified from Klopfstein & Spasojevic, 2019)
(0) 1-0.6 (more or less parallel);
(1) <0.6 (strongly converging ventrally).

**#23 Eyes, lateral view, height relative to head height [ordered]:** (Klopfstein & Spasojevic, 2019)
Ratio of eye height (take highest measure) in lateral aspect to head height (from the base of mandible to the highest visible point in lateral aspect).

**#24 Frontal orbit, colour of female:** (Klopfstein & Spasojevic, 2019)
(0) concolorous with remainder of frons;
(1) with colour markings, different from head ground colour.

**#25 Face, eyes, surface:** (Klopfstein & Spasojevic, 2019)
(0) with no or at most some inconspicuous hairs;
(1) with long, thin hairs that are conspicious (at least when viewed against a dark background).

**#26 Head, ocelli, size:** (Klopfstein & Spasojevic, 2019)
(0) of normal size, separate from each other and eyes by more than their diameter, on one plane with vertex;
(1) enlarged, closely spaced, on raised triangular area.

**#27 Head, gena (area behind eyes), sculpture:** (Klopfstein & Spasojevic, 2019)
(0) smooth, punctured or shagreened;
(1) with scale-like denticles.

**#28 Head, posterior side, occipital carina, upper end, form:** (modified from Klopfstein & Spasojevic, 2019)
(0) more or less complete, dorsally evenly convex or slightly flattened, normally raised;
(1) broadly absent dorsally, but ventrally present;
(2) entirely absent;
(3) more or less complete, dorsally evenly convex, flange-like;
(4) more or less complete, dorsally strongly dipped, weakly raised, sometimes very weak or obsolescent near midline, but then with upper end of lateral portion curved downwards;
(5) complete, with a median notch (Dyspetes).

**#29 Head, profile view, vertex:** (modified from Klopfstein & Spasojevic, 2019)
(0) moderately long, weakly and evenly rounded down to occipital carina;
(1) steeply declivous behind ocelli but surface flat or slightly convex;
(2) long and convex, usually with occipital carina rather low on head;
(3) precipitously declivous behind posterior ocelli, concave;
(4) raised or horizontal, then abruptly declivous behind occipital carina;
(5) like 1 but occipital carina very low on head;
(6) very short, distance between lateral ocelli and occipital carinae less than the diameter of the ocelli (cf. Anomaloninae);
(7) horizontal to occipital carina and weakly convex (cf. Allomya)

**#30 Head, frons, deep impressions that accomodate scape: (Meier et al. 2022)**(0) absent;
(1) present.

**#31 Antennae, apical flagellomere modification (female) [associated with echolocation]:** (split, modified from Klopfstein & Spasojevic, 2019)
(0) absent;
(1) present.

**#32 Antennae, apical flagellomere, type of modification (female):** (split, modified from Klopfstein & Spasojevic, 2019)
(0) with some thickened setae;
(1) with cluster of modified setae;
(2) dense cluster of thicked and truncate structures froming a patch, no fusion of the structures, fused in the middle, or forming a flat surface;
(3) the flagellomer itself with apex pointed;
(4) apex of flagellomere truncated to leave a flat surface that is devoid of sensillae or hairs (cf. Echthrus);
(5) with two rows of conspiciously modified setae along entire length of apical flagellomere (cf. Xorides).

**#33 Antenna, pedicle, size:** (Klopfstein & Spasojevic, 2019)
(0) clearly smaller than scape, not inflated, and only of slightly greater diameter than 1st flagellomere (Gauld ea 2002: Fig. 33);
(1) strongly inflated, almost as broad as scape and distinctly broader than 1st flagellomere (Gauld ea 2002: Fig. 34).

**#34 Antenna, scape, shape [ordered]:** (Klopfstein & Spasojevic, 2019)
(0) conspicuously shortened, so it is laterally no longer than pedicel and shorter than wide (cf. Metopius);
(1) 1-1.5 times longer than wide, truncation oblique;
(2) elongate-cylindrical, >1.8x as long as wide, truncation at about 60-90° to longitudinal axis (cf. Orthocentrus group);

**#35 Antenna, scape, extended membranous area:** (Klopfstein & Spasojevic, 2019)
(0) developped normally;
(1) conspiciously expanded on dorsal side (cf. Megastylus);
(2) expanded ventrally, with dorsal side of scape inflexed under the ventral side (cf. Xanthopimpla rhopalocera).

**#36 Antenna, flagellum, colour:** (Klopfstein & Spasojevic, 2019)
(0) without a median white band;
(1) with a median white band;
(2) with a basal white band (e.g. Cymodusa).

**#37 Antenna, flagellum, basal or central flagellomeres, tyloids, male:** (split, modified from Klopfstein & Spasojevic, 2019)
(0) absent;
(1) present:

**#38 Antenna, flagellum, basal or central flagellomeres, tyloid shape, male:** (split, modified from Klopfstein & Spasojevic, 2019)
(0) elliptical (cf. Sussaba cognata);
(1) linear (cf. Promethes sulcator);
(2) concave (Cylloceriinae);
(3) raised, tooth-like (cf. Helcostizus restaurator).

**#39 Antenna, length related to fore wing length [ordered]:** (Klopfstein & Spasojevic, 2019)
Ratio

**#40 Antenna, number of flagellomeres [ordered]:** (Klopfstein & Spasojevic, 2019)
Ratio

**#41 Antenna, thickness:** (Klopfstein & Spasojevic, 2019)
(0) more or less of even thickness;
(1) evenly tapering from apex to base, broadest flagellomeres close to antennal tip;
(2) abruptly enlarged close to apex, including only a handful of segments;
(3) tapering towards apex, last segments conspicuously narrower than basal ones;
(4) broadest in the middle, tappering from there towards base and apex (cf. Euceros).

**#42 Antenna, dimensions of 1st flagellomere [ordered]: (Meier et al. 2022)**
Ratio - length of flagellomer 1 to apical width ratio.

**#43 Frons, modifications between antennal sockets and median ocellus:** (split, modified from Klopfstein & Spasojevic, 2019)
(0) absent;
(1) present.

**#44 Frons, modifications type:** (split, modified from Klopfstein & Spasojevic, 2019)
(0) strongly raised keel between antennae, reaching past antenna posteriorly, stops posteriorly to antenna (Metopiinae);
(1) two low, parallel ridges from inner side of antennal scrobes towards middle ocellus;
(2) rather low longitudinal ridge that might expand into a lobe at level of antennal sockets and reaching at least to ventral margin of antennal sockets (Acanitinae);
(3) raised keel only between antenna that continues as a low ridge towards lower face (the dorsal part same shape as in state 0);
(4) strong impression between toruli and behind each toruli on the side closer to eyes, the central impression separated from the side ones by oblique ridges;
(5) with a horn, which can be hollowed from above.
(6) impressions for scapus, in between two projecting processes with pointed ends;
(7) longitudinal keel between two impressions (Townesitinae);
(8) two high lobes behind the antennal sockets (Rogichneumon braconicus);
(9) strongly raised keel between antennae, reaching past antenna posteriorly, continues as low ridge to median ocellus (Metopiinae:Triclistus, Cubus, Colpotrochia).

**#45 Pronotum, lateral view, shape [ordered]:** (Klopfstein & Spasojevic, 2019)
(0) short and deep, <0.6 times as long as deep, with collar (if present) very close to mesoscutal margin (Fig. 48);
(1) moderately long, 0.8–1.0 times as long as deep, collar not close to mesoscutum (Figs 47, 52);
(2) long, >1.1 times as long as deep, collar not close to mesoscutum but mediodorsal region still rather short and furrowed (Fig. 50);
(3) long, but with mediodorsal region horizontal and lengthened (Fig. 51).

**#46 Pronotum, anterior margin:** (Klopfstein & Spasojevic, 2019)
(0) simple; (1) mediodorsally reflexed, and directed backwards as a strong pointed tubercle (Figs 47, 48, 50); (2) with a very weak posteriorly directed median indentation.

**#47 Pronotum, posterior part near midline:** (modified from Klopfstein & Spasojevic, 2019)
(0) vertical part not or only slightly thickened, horizontal part simple;
(1) raised as a distinct horizontal prominence, but this prominence still fused with remainder of pronotum;
(2) with horizontal ‘shelf ’not laterally closed or laterally closed to form a pocket-like structure (Fig. 51);
(3) with one median and two lateral ridges (in addition to the epomia - compare *Xorides praecatorius*);
(4) with one median ridge (see Poecilocryptus);
(5) vertical part not or only slightly thickened, horizontal part thickned anterior and with clear transversal impression posterior;
(6) longitudinally impressed forming a channel (cf. Rhyssa persuasoria);
(7) with a longitudinal chanel plus thickened anteriorly and posteriorly with transverse impression (Diacritus);
(8) with two lateral longitudinal ridges enclosing triangular area (Braconidae: Homolobus);
(9) with a triangular impression at the base of vertical part; vertical part simple.

**#48 Pronotum, epomia:** (Klopfstein & Spasojevic, 2019)
(0) complete, forming a weak ridge shaped like a figure 7 or reduced with only upper part present; (1) entirely absent (Figs 49, 51);
(2) with lower part long, extending almost till the ventral margin of the pronotum, while pronotum clearly rised below the epomia;
(3) with lower part long and strongly raised as a flange;
(4) with epomia very strong and expanded dorsally into a tooth;
(5) as 2 but pronotum not raised below the epomia, instead the anterior margin protruding into a tooth.

**#49 Pronotum, upper hind corner [ordered]:** (Klopfstein & Spasojevic, 2019)
(0) with a tiny sclerite present between hind corner of pronotum, mesopleuron and tegula, thus causing the pronotum to bow out a little around this sclerite (prepectus? drawn on page 44 of Townes vol 1, but not named);
(1) with hind corner of pronotum overgrowing this sclerite, but sclerite partially visible (e.g. Banchinae, Ctenopelmatinae);
(2) with hind corner of pronotum overgrowing this sclerite, sclerite completely overgrown and invisible (cf. Metopiinae)

**#50 Propleuron, posterior side, lobe:** (Klopfstein & Spasojevic, 2019)
(0) absent (Fig. 2);
(1) present (Wahl Fig. 2-33 Venturia sokanakiakorum);

**#51 Mesoscutum, sculpture:** (modified from Klopfstein & Spasojevic, 2019)
(0) finely punctate;
(1) smooth and impunctate;
(2) very coarsely punctate, with punctures separated inter se by about their own diameter;
(3) coarsely and closely punctate, the punctures more or less contiguous;
(4) evenly shagreened and matt;
(5) with sharp transverse rugae;
(6) with strong punctures and longitudinal striae formed of confluent punctures (cf. Armadillocryptus);
(7) with coarse rugae forming a net-like pattern, at least on median part.

**#52 Mesoscutum, pubescence:** (Klopfstein & Spasojevic, 2019)
(0) evenly pubescent;
(1) glabrous, at most with a few hairs around front or position of notauli, these hairs short;
(2) glabrous, but with a few very long hairs along front and position of notauli, these hairs as long as diameter of flagellomere 1;
(3) middle evenly pubescent, laterally more glabrous.

**#53 Mesoscutum, carina along lateral margin:** (Klopfstein & Spasojevic, 2019)
(0) complete to anterior end of scutellum;
(1) evanescent, not reaching the scutellum.

**#54 Mesoscutum, lateral carina, posteriorly, height:** (Klopfstein & Spasojevic, 2019)
(0) normal carina, not or only barely broader than it is anteriorly;
(1) strongly broadened, at least twice as wide as it is anteriorly.

**#55 Mesoscutum, near base of notaulus, modification:** (split, modified from Klopfstein & Spasojevic, 2019)
(0) absent;
(1) present.

**#56 Mesoscutum, near base of notaulus, modification, type:** (split, modified from Klopfstein & Spasojevic, 2019)
(0) with a thickening or crest at the base of notauli (cf. Lissopimpla, Xanthopimpla);
(1) with a paired crest at the  anterior dorso-lateral part of propodeum (cf. Acrodactyla degener);
(2) with a short carina directly in front of and parallel to notaulus (cf. some Orthocentrinae);
(3) with pronotal margine laterally swollen.

**#57 Mesoscutum, notaulus, strength [ordered]:** (Klopfstein & Spasojevic, 2019)
(0) absent;
(1) shallow or vestigial;
(2) deeply impressed at least anteriorly.

**#58 Mesoscutum, notaulus:** (Klopfstein & Spasojevic, 2019)
(0) present only on the frontal vertical part of mesoscutum
(1) extending to half length of mesoscutum, more or less parallel;
(2) extending to half length of mesoscutum, strongly converging;
(3) extending posteriorly past centre of mesoscutum but not joining other notaulus;
(4) extending posteriorly past centre and joining either the other notaulus or a sunken, medial area (Fig. XX Diacritus);
(NA) absent.

**#59 Scutellum, lateral longitudinal carinae, completeness:** (Klopfstein & Spasojevic, 2019)
(0) absent or only discernible on extreme anterior end;
(1) more or less complete to centre or beyond, but not reaching posterior margin;
(2) complete to posterior margin;
(3) complete AND expanded posteriorly into two acute teeth (cf. Metopius).

**#60 Scutellum, lateral view, shape:** (Klopfstein & Spasojevic, 2019)
(0) more or less flat;
(1) a bit convex;
(2) pyramidal = very strongly convex in profile, sometimes even drawn into a point between base and middle of scutellum (more triangular pyramid), posterior surface long, evenly declivous;
(3) strongly convex in profile, posterior surface convex, rather short (e.g. Phorotrophus);
(4) pyramidal in profile, but with acute highest point almost at the end, after steeply declivous (Astiphromma dorsale group); (5) as state 2, but with carinae along the posterior slope (and at lower front corners - triangular pyramid);
(6) strongly pyramidal in profile, but also with the whole shape (Opheltes);
(7) bit convex convex with posterior part raised (Tymmophorus).

**#61 Metanotum (=postscutellum): shape:** (modified from Klopfstein & Spasojevic, 2019)
(0) normally flat to evenly convex;
(1) conspiciously short, sharply rounded and strongly declivous posteriorly (cf. Camptotypus);
(2) triangular in profile and pointed (cf. some Orthocentrinae);
(3) long and flat, with two low longitudinal carinae and small swelling posteriorly;
(4) nearly absent, only a slightly thickened carina in its place (cf. Diaparsis).

**#62 Scutellum, metanotum/metapleuron, length:** (modified from Klopfstein & Spasojevic, 2019)
(0) short, so that the constriction between metanotum and propodeum is very close after the postscutellum;
(1) elongate, so that the constriction is clearly removed from the postscutellum, by about 0.2-0.3x the length of the propodeum.

**#63 Mesopleuron centrally:** (modified from Klopfstein & Spasojevic, 2019)
(0) rather flat or weakly convex, at most with a weak diagonal impression delineating a convex area posterodorsally (Fig. 54);
(1) with a strong horizontal impression reaching from mesopleural sulcus forward to near upper end of epicnemial carina;
(2) very strongly convex so that the body is much wider when viewed from above at height of mesopleuron than further back at the mesepimeron (cf. Metopius);
(3) horizontal groove from lower third of hind margin of pronotum to clearly below mesosternal scrobe.

**#64 Mesopleuron, epicnemial carina, shape:** (Klopfstein & Spasojevic, 2019)
(0) not curving anteriorly, vertical to around mid-height of pronotum (Fig XX Diacritus);
(1) curving to anterior end of mesopleuron at around the mid-height of the pronotum (Fig. XX Rhimphoctona);
(2) extending all the way to subtegular ridge (Fig. 2);
(3) present ventrally only (not extending dorsally to ventral edge of pronotum);
(4) present only laterally;
(5) absent.

**#65 Mesopleuron, mesopleural furrow:** (Klopfstein & Spasojevic, 2019)
(0) angled opposite mesepisternal scrobe, usually with a shallow horizontal impression extending from this angulation to the scrobe (Fig. 53);
(1) more or less straight with a shallow horizontal impression extending from this angulation to the scrobe;
(2) more or less straight with isolate punctute instead of the horizontal impression (Fig. 54).

**#66 Mesopleuron, ventrally (mesosternum), behind fore coxae:** (Klopfstein & Spasojevic, 2019)
(0) simply transverse;
(1) produced forwards in a broad blunt angulation (anterior transverse carina produced forward);
(2) produced forwards in a narrow blunt angulation, with mesosternum protruding in the middle forming a vertical ridge;
(3) produced forward into a large lobe;
(4) with a pointed tubercule in the middle of mesosternum.

**#67 Mesopleuron, ventrally, posterior transverse carina of mesosternum:** (modified from Klopfstein & Spasojevic, 2019)
(0) absent;
(1) absent in front of the middle coxae, present otherwise (Fig. xx, Therion);
(2) complete (Fig. xx, Dusona);
(3) short and present only in the middle in-between the two mid coxae;
(4) clearly present laterally, but absent in front of the mid coxa, in between coxae absent/fused with anterior transverse carina;
(5) absent laterally but present as extended flanges in front of the mid coxae and in between;
(6) absent laterally but present as extended flanges in front of the mid coxae, otherwise normal carinae between middle coxa

**#68 Mesopleuron, laterally, sternaulus:** (Klopfstein & Spasojevic, 2019)
(0) Absent or present less than 0.7x length of mesopleuron;
(1) present, greater than or equal to 0.7x length of mesopleuron

**#69 Mesopleuron, laterally, posterior ending of sternaulus:**(0) ending below or at level of mid coxa (many Cryptinae);
(1) ending above level of mid coxa (many Phygadeuontinae).

**#70 Metapleuron, dimensions [ordered]:** (Klopfstein & Spasojevic, 2019)
(0) clearly higher than long (cf. Diplazontinae);
(1) about as long as wide;
(2) 1.7-2x as long as wide.

**#71 Metapleuron, submetapleural carina:** (Klopfstein & Spasojevic, 2019)
(0) complete, anterior section unmodified to slightly broadened (Fig xx Pimpla);
(1) complete, anterior section abruptly broadened into a lobe (Fig. xx. Exetastes fornicator…);
(2) anteriorly present, not-lobe like, posteriorly absent (Fig. xx Dolichomitus);
(3) completely absent;
(4) as 0,1 but not complete to posterior edge;
(5) complete, broadened on the whole length.

**#72 Propodeum, pleural carina, presence [ordered]:** (Klopfstein & Spasojevic, 2019)
(0) mostly absent (only with indications at beginning and/or end);
(1) partially reduced or weak;
(2) present as distinct carina on whole length.

**#73 Propodeum, juxacoxal carina, presence [ordered]:** (Klopfstein & Spasojevic, 2019)
(0) mostly absent (only with indications at beginning and/or end);
(1) partially reduced or weak;
(2) present as distinct carina on whole length.

**#74 Propodeum, lateral view, shape:** (modified Klopfstein & Spasojevic, 2019)
(0) from rounded to dorsally flattened, and with a posterior surface (Fig. 59);
(1) evenly declivous (Fig. 60);
(2) with postero-medial part elongated behind the hind coxae into a process that carries the metasoma, this process about 1/3 to 2/3 as long as propodeum itself;
(3) with petiolar area deeply concave to take in petiolus (cf. Townesitinae, maybe Sinophorus?).

**#75 Propodeum, dimension [ordered]:** (Klopfstein & Spasojevic, 2019)
(0) clearly shorter than high (cf. Diplanzontinae);
(1) about as long as high (Figs 61, 62) (but sometimes with postero-median part further elongated, cf. Heteropelma);
(2) elongate, distinctly longer than wide, cylindrical;

**#76 Propodeum, longitudinal carinae, presence:** (Klopfstein & Spasojevic, 2019)
(0) absent;
(1) only lateromedian longitudinal carinae present;
(2) only lateral longitudinal carinae present;
(3) both lateromedian and lateral longitudinal carinae present.

**#77 Propodeum, anterior transverse carina, completeness:** (Klopfstein & Spasojevic, 2019)
(0) complete (medially and sublaterally) (Fig. 3);
(1) medial abscissa present, sublateral abscissae (= costulae) absent (Fig. xx Aplomerus tibialis scan); (2) median abscissae absent, sublateral abscissae present (Fig. xx Ateleute tsiriria scan);
(3) completely absent.

**#78 Propodeum, anterior transverse carina, shape:** (Klopfstein & Spasojevic, 2019)
(0) angled (Fig. 3);
(1) forming more or less a smooth arc (Fig. xx Ophion flavidus). Note: if carina absent or incomplete so that angulation/curvature could not be scored, characters is coded as NA

**#79 Propodeum, posterior transverse carina, completeness:** (Klopfstein & Spasojevic, 2019)
(0) complete (medially and sublaterally) (Fig. 3);
(1) medial abscissa present, sublateral abscissae absent;
(2) median abscissa absent, sublateral abscissae present (areolar area confluent with petiolar area) (Fig. xx Campoplex hyalinus);
(3) completely absent/ indistinguishable.

**#80 Posterior transverse carina, shape:** (Klopfstein & Spasojevic, 2019)
(0) angled (Fig. 3);
(1) more or less a continuous arc (Fig. xx Lissonota catenator).

**#81 Spiracle, shape [ordered]:** (Klopfstein & Spasojevic, 2019)
(0) subcircular to oval, < 2.0 times as long as wide (Fig. 59);
(1) elongately oval >2.0 but less than 3.5 times as long as wide (Fig. 60);
(2) elongately elliptical >3.5 times as long as wide (Fig. 63).

**#82 Spiracle, distance separated from pleural carina:** (Klopfstein & Spasojevic, 2019)
(0) separated from pleural carina by about minimum diameter of spiracle or more (Fig. 2),
(1) separated from pleural carina by less than minimum diameter.

**#83 Posterolateral angle:** (Klopfstein & Spasojevic, 2019)
(0) evenly rounded;
(1) swollen (Fig. 59, Zaglyptus).

**#84 Modifications at junction of lateral longitudinal and posterior (apical) transverse carinae:** (Klopfstein & Spasojevic, 2019)
(0) absent (Fig. 56);
(1) with subconical apophyses (Figs 57, 63);
(2) with posterior transverse carina elevated into a flange on points where lateral longitudinal carina would have joined (cf. Buathra);
(3) with flanges (cf. Opheltes).

**#85 Modifications on postero-median surface of propodeum:** (Klopfstein & Spasojevic, 2019)
(0) without modifications;
(1) with a strong single median tubercle (Figs 57, 63);
(2) with posterior transverse carina elevated into a flange that forms a shelf;
(3) with a deep impression that accomodates T1 (some Phygadeuontinae).

**#86 Propodeum, hind margin:** (Klopfstein & Spasojevic, 2019)
(0) simple;
(1) extended and covering anterior part of metasomal first tergit.

**#87 Propodeum, sculpture:** (Klopfstein & Spasojevic, 2019)
(0) smooth and shiny, without reticulation between carinae and at most with some weak punctures where hairs are inserted (Fig. 55*);
(1) mostly covered by reticulation that obscures carinae;
(2) with distinct punctures;
(3) rugulopunctate;
(4) with weak punctures, distance more than their diameter;
(5) evenly shagreened and matt without distinct punctures;
(6) with strong wavy pattern between carinae (cf. Armadillocryptus).

**#88 Metasomal foramen ventrally, shape:** (Klopfstein & Spasojevic, 2019)
(0) ventrally broadly U-shaped to a somewhat rounded V-shape;
(1) deeply V-shaped.

**#89 Metacoxal cavity [ordered]:** (Klopfstein & Spasojevic, 2019)
(0) dorsal margin is dorsal to the ventral margin of metasomal foramen magnum;
(1) dorsal margin just on height of lower margin of metasomal foramen magnum;
(2) dorsal margin is (clearly) ventral to the ventral margin of metasomal foramen magnum.

**#90 Intercoxal carinae:** (Klopfstein & Spasojevic, 2019)
(0) weak, usually with vestiges meeting ventral rim of metasomal foramen;
(1) with posterior end of carina meeting its fellow medially and forming a short but strongly raised medial carina that reaches back to the rim of the foramen (Fig. 87);
(2) abruptly angled posteriorly to meet its fellow and not joined to rim of foramen, instead making a sharp carina between or in front of coxal foramena.

**#91 Hind coxa, podeme:** (Klopfstein & Spasojevic, 2019)
(0) with lateral apodeme not or barely twisted;
(1) with lateral apodeme twisted about 60–75°, and with a corresponding deep notch in lateral margin of coxal socket (cf. Fig.63 which has a notch with Fig. 61 where one is not present).

**#92 Propodeal denticles:** (Klopfstein & Spasojevic, 2019)
(0) absent;
(1) present (Fig. 64).

**#93 Fore legs, femur, female:** (Klopfstein & Spasojevic, 2019)
(0) ventrally unspecialized, more or less convex or slightly flattened;
(1) with a longitudinal concavity (which may sometimes be biconcave).

**#94 Fore legs, tibia, shape, female:** (Klopfstein & Spasojevic, 2019)
(0) simple;
(1) enlarged and inflated;
(2) slender and bowed to fit around angularly produced femur.

**#95 Fore legs, tibia, spines (not setae):** (Klopfstein & Spasojevic, 2019)
(0) without spines;
(1) with scattered long and stronger spines;
(2) bearing stout short conical spines set in pits.

**#96 Fore legs, apex of tibia, tooth:** (Klopfstein & Spasojevic, 2019)
(0) without tooth;
(1) with a distinct tooth on dorsal margin.

**#97 Fore legs, 4th tarsomere, dimension: (Alvarado Gutiérrez, 2018)**(0) longer than wide;
(1) wider than long or about as long as wide.

**#98 Fore legs, 5th tarsomere, female:** (Klopfstein & Spasojevic, 2019)
(0) slender, of similar thickness to other tarsomeres;
(1) exceptionally swollen.

**#99 Fore legs, femur length (incl. trochanter) compared with forewing:** (Klopfstein & Spasojevic, 2019)
(0) < 0.3x forewing;
(1) > 0.3x forewing.

**#100 Fore and mid legs, trochantellus, fusion: (Alvarado Gutiérrez, 2018)**(0) clearly differentiated from femur;
(1) fused.

**#101 Mid coxa, inner surface, female:** (Klopfstein & Spasojevic, 2019)
(0) simply convex;
(1) with shallow groove;
(2) with shallow groove bordered by sharp parallel carinae.

**#102 Mid coxa, outer surface, female:** (Klopfstein & Spasojevic, 2019)
(0) simply convex, unspecialized;
(1) with convex impressions so it appears to have bumps.

**#103 Mid tibia, number of spurs:** (Klopfstein & Spasojevic, 2019)
(0) two spurs;
(1) one spur.

**#104 Mid tibia, inner spur, relative length to outer spur [ordered]: (Alvarado Gutiérrez, 2018)**(0) inner spur longer than outer spur;
(1) both spurs equal in length;
(2) outer spur longer than inner spur.

**#105 Mid and hind tibia, ventral view, apex:** (Klopfstein & Spasojevic, 2019)
(0) with common area of insertion for spurs and basitarsus;
(1) with sclerotized bridge separating insertion areas (Fig. xx Eiphosoma).

**#106 Hind coxa, proportions [ordered]:** (Klopfstein & Spasojevic, 2019)
(0) short and strongly inflated, deeper than long;
(1) of normal dimensions, slightly longer than deep;
(2) elongate and slender, 1.5-3 times as long as deep;
(3) extremely elongate, > 3.5x as long as deep.

**#107 Hind coxa, posterior side, modification:** (modified from Klopfstein & Spasojevic, 2019)
(0) evenly rounded;
(1) bluntly angled on ventrolateral edge;
(2) with a tooth on under side towards the apex (cf. Phaeogenes).

**#108 Hind coxa, inner surface, female:** (Klopfstein & Spasojevic, 2019)
(0) simply convex;
(1) with shallow groove;
(2) with shallow groove bordered by sharp parallel carinae;
(3) with furrow covered with grossulariform setae.

**#109 Hind femur, ventral, modification:** (Klopfstein & Spasojevic, 2019)
(0) simple;
(1) with a blunt tooth.

**#110 Hind femur, dimensions [ordered]:** (Klopfstein & Spasojevic, 2019)
(0) conspicuously thickened, up to 2, 5 time as long as deep;
(1) normal dimensions, between three and five times as long as deep;
(2) elongate, more than 5x as long as deep.

**#111 Hind tibia, spurs, dimension:** (Klopfstein & Spasojevic, 2019)
(0) long and slender, of normal dimensions, pointed apically;
(1) short and stout, the inner spur with a broad, truncated edge;
(2) short and stout, but with tips normal, even though often quite curved;
(3) inner long and slender, outer short and very stout;
(4) very short and slender. (NA if one or zero spurs)

**#112 Hind tibia, number of spurs [ordered]: (Alvarado Gutiérrez, 2018)**
(0) two spurs;
(1) one spur;
(2) no spurs.

**#113 Hind tibia, pubescence:** (Klopfstein & Spasojevic, 2019)
(0) covered with a uniform thickness of setae;
(1) with uniform setae and sparse, stouter spines (spines much slender compared to the state 2 spines, not very obvious among setae);
(2) with uniform setae and sparse, very stout conus shaped spines (spines very conspicous, setae very inconspicuos)

**#114 Hind tibia, colouration:** (Klopfstein & Spasojevic, 2019)
(0) uniformly red, dark brown, orange or yellow;
(1) orange or brown with apex darkened;
(2) white with apex darkened;
(3) black-white-black banded;
(4) orange or yellow with base and apex darkened;
(5) red, brown, orange with base lighter;
(6) black with a narrow light base;
(7) orange or yellow with base darkened;
(8) longitudinally split: orange in posterior, brown in anterior half.

**#115 Hind tibia, shape:** (Klopfstein & Spasojevic, 2019)
(0) normal, parallel sided to evenly tapered or a bit enlarged, so that base is distinctly thinner;
(1) very strongly enlarged, becoming tubular;
(2) narrowing in the middle.

**#116 Hind tibia, apex, inner side, fringe of parallel setae:** (Klopfstein & Spasojevic, 2019)
(0) absent, only with a few hairs apically which are very similar as the hairs on the surface;
(1) present as a fringe of parallel setae which are very similar to the main pubescence on the hind tibia;
(2) present as a very dense fringe of parallel bristle which clearly differ from the main pubescence of the tibia AND from the fringe on the OUTER surface.

**#117 Hind tibia, 1st tarsomere, dimensions [ordered]:** (Klopfstein & Spasojevic, 2019)
Ratio - write down the proportions.

**#118 Hind leg, 2nd to 4th tarsomere of female ventrally:** (Klopfstein & Spasojevic, 2019)
(0) with pubescence that is similar to the dorsal pubescence;
(1) with strong stout spines that are obviously thicker than the dorsal pubescence (Fig. 92).

**#119 Hind leg, 4th hind tarsomere:** (Klopfstein & Spasojevic, 2019)
(0) apically more or less evenly truncated;
(1) apically obliquely truncated, so that inner side overhangs base of the fifth tarsomere.

**#120 Tarsal claws, modification (female):** (split from Klopfstein & Spasojevic, 2019)
(0) absent;
(1) present.

**#121 Tarsal claws, modification (female):** (modified from Klopfstein & Spasojevic, 2019)
(0) all modified;
(1) hind simple, others modified;
(2) fore modified others simple.

**#122 Tarsal claws, modification, location (female):** (Klopfstein & Spasojevic, 2019)
(0) broad basal lobe;
(1) additional tooth next to the tip of the claw;
(2) claws pectinate, i.e., with a comb-like row of teeth starting near base and extending to middle or even to apex of claw; (NA) = claws simple

**#123 Hind claws, apex:** (Klopfstein & Spasojevic, 2019)
(0) simply pointed;
(1) bevelled.

**#124 Hind claws, orbicula (sclerotized part between claws), form:** (modified from Klopfstein & Spasojevic, 2019)
(0) short and moderately broad, between 2.5 and 3.5 times as long as wide;
(1) very slender and bar-like, >3.5 times as long as wide (Fig. 91);
(2) very short, only about 2x as long as wide.;
(3) broad and expanded anteriorly, almost as long as wide (see Heteropelma)

**#125 Hind claws, arolium (membranous pad between claws), size:** (Klopfstein & Spasojevic, 2019)
(0) of normal dimensions, not projecting beyond apex of claw;
(1) enlarged, projecting conspicuously beyond apex of claw (Fig. 93).

**#126 Hind claws, inner side, poison vesicle:** (Klopfstein & Spasojevic, 2019)
(0) without any trace of a membranous poison vesicle;
(1) with a small membranous vesicle on inside of apical tooth of male;
(2) with a small membranous vesicle in male and female;
(3) with a large membranous vesicle in male and female.

**#127 Hind claws, spatulate bristle:** (Klopfstein & Spasojevic, 2019)
(0) without a spatulate bristle;
(1) with a long conspicuous spatulate bristle, the apex of which (at rest) is pressed against the pointed apex of the claw.

##################################################################################

Wings

**#128 Fore wing, areolet, vein 3rs-m:** (Klopfstein & Spasojevic, 2019)
(0) absent;
(1) spectral ~ 100%;
(2) 1 bulla;
(3) 2 bullae;
(4) without bulla.

**#129 Fore wing, areolet, shape:** (Klopfstein & Spasojevic, 2019)
(0) quadrate-oblique to triangular, with 2+3M longer than 4M, 2Rs more than 0.3x 3M;
(1) rombic, all sides almost equal;
(2) pentagonal, with +/- even sides;
(3) as 0, but 2Rs less than 0.3x 3M (note if all obliterated);
(4) widened pentagonal, > 1.5 times as wide as deep (Labenopimplinae type),
(5) Braconidae condition, not proper areolet, but cell that is of similar size to other cells (widened quadrate, 2+3M and 4M together, 2m-cu absent, 3Rs long);
(6) strongly petiolate areolet (put <= one vein diameter into state 0 or 9),
(7) quadrate? with 2Rs more than 2x 2+3M;
(8) with 2Rs absent, thus areolet open towards the inside;
(9) quadrate-asymmetric, but not oblique: with 3M shorter than 4M, and 2Rs of similar length as 3M (usually rather big areolet, cf. many Metopiinae)

**#130 Fore wing, vein 2m-cu, presence:** (modified from Klopfstein & Spasojevic, 2019)
(0) present (Fig. 4);
(1) absent;
(2) indicated by angleation of 4Rs and/or 5M

**#131 Fore wing, vein 2m-cu, number of bullae:** (Klopfstein & Spasojevic, 2019)
(0) with two bullae separated with only trace of the vein or with longer part of the vein;
(1) with a single bulla;
(2) entirely spectral. [Comment SK: add state for 2 bullae, but both above middle -> cf. Labenini]

**#132 Fore wing, vein 2m-cu, size of bullae [ordered]:** (Klopfstein & Spasojevic, 2019)
Percent of the vein they are covering (eg. When 1: 80, when 2: 20+20).

**#133 Fore wing, vein 2m-cu, position of bullae: NEW**(0) rather anteriorly;
(1) eveny distributed or centred;
(2) rather posteriorly on vein.

**#134 Fore wing, vein 2m-cu, shape:**(0) straight;
(1) more or less evenly bowed outwards;
(2) anteriorly bowed outwards, posteriorly inwards, thus forming a zig-zag or at least sinuous curve; (3) bowed or angled inwards;
(4) anteriorly straight or even bowed inwards, about posterior half bowed outwards.

**#135 Fore wing, length of 4Cu relative to 5Cu [ordered]: NEW**
Calculation: 4Cu/(4Cu+ 5Cu).

**#136 Fore wing, discosubmarginal cell (1M+1R1):** (Klopfstein & Spasojevic, 2019)
(0) uniformly hirsute;
(1) with small to large glabrous area that may have free sclerites (cf. Leptophion).

**#137 Fore wing, vein 4Rs, shape:** (Klopfstein & Spasojevic, 2019)
(0) almost straight;
(1) proximaly arched;
(2) distally arched;
(3) clearly sinusoidal;
(4) evenly arched towards 1R1;
(5) evenly arched towards 2R1.

**#138 Fore wing, ramulus (remaining of the vein 1Rs+M) [ordered]:**(0) absent;
(1) present, at most as long as the width of the surrounding veins;
(2) present, longer as the width of the surrounding veins (mostly reaching till half of the way to M).

**#139 Fore wing, vein 1cu-a (nervullus position) [ordered]:** (Klopfstein & Spasojevic, 2019)
(0) meeting vein M+CU basal of M&RS by more than 1 vein width ("nervulus antefurkal");
(1) at level of M&RS ("interstitial");
(2) distal of M&RS by more than 1 vein width ("postfurkal");
(3) extremelly posterior so that vein 1Cu is more than 0.8x longer than the vein 2Cu (some Brachycyrtinae)

**#140 Fore wing, pterostigma, dimensions [ordered]:** (Klopfstein & Spasojevic, 2019)
Write ratio length to height.

**#141 Fore wing, ratio of pterostigma length to 1R1 (vein) length [ordered]:** (Klopfstein & Spasojevic, 2019)
Write ratio.

**#142 Fore wing, marginal cell (2R1), dimensions [ordered]:** (Klopfstein & Spasojevic, 2019)
Write ratio.

**#143 Fore wing, vein 5M [ordered]:** (Klopfstein & Spasojevic, 2019)
(0) vein tubular through the whole length (not counting the last 10% of the vein), same colour as or slightly lighter than the colour of the surrounding veins;
(1) vein partly spectral (not counting the last 10% of the vein);
(2) vein throught whole lenght spectral (+-);
(3) absent.

**#144 Fore wing, ratio of 2Cu length to 1M+1Rs length [ordered]:** (Klopfstein & Spasojevic, 2019)
Write ratio.

**#145 Fore wing, ratio of 2Cu length to r-rs length [ordered]:** (Klopfstein & Spasojevic, 2019)
Write ratio.

**#146 Fore wing, ratio of 1M+1Rs to r-rs [ordered]: (Meier et al. 2022)**Write ratio.

**#147 Fore wing, 1m-cu&2Rs+M vein, shape:** (Klopfstein & Spasojevic, 2019)
(0) straight;
(1) arched;
(2) angled;
(3) with a rounded hump;
(4) distal bow very strong.

**#148 Fore wing, postnervellus, location of the junction from vein 2Cu-a and 3Cu to vein 4Cu [ordered]:** (Klopfstein & Spasojevic, 2019)
(0) 0-0.25;
(1) 0.25-0.5;
(2) 0.5-0.75;
(3) 0.75-1.

**#149 Vein M+CU, shape:** (Klopfstein & Spasojevic, 2019)
(0) straight, except for short bow near base;
(1) curved distally;
(2) curved at round middle, more or less straight before and after;
(3) slightly curved on entire length.

**#150 Vein M+Cu, completeness:** (Klopfstein & Spasojevic, 2019)
(0) complete;
(1) basal 0.6 spectral (Fig. xx Allophrys)

**#151 Hind wing, 2nd abscissa of CU (2CU) / (2CU + cu-a) (nervellus), interception [ordered]:** (Klopfstein & Spasojevic, 2019)
(0) 0-0.25;
(1); 0.25-0.5;
(2) 0.5-0.75;
(3) 0.75-1; NA- inapplicable, nervellus not intercepted. [comment SK: add option for discoidella attaching to cubitella instead -> cf. Apechoneura].

**#152 Hind wing, last abscissa of RS (2RS), sclerotization [ordered]:** (Klopfstein & Spasojevic, 2019)
(0) vein tubular through the whole length (not counting the last 10% of the vein), same colour as or slightly lighter than the colour of the surrounding veins;
(1) vein partly spectral (not counting the last 10% of the vein);
(2) vein throught whole lenght spectral (+-);
(3) absent (not counting the first and last 10% of the vein, even though they can be tubular).

**#153 Hind wing, last abscissa of CU (2CU), sclerotization [ordered]:** (Klopfstein & Spasojevic, 2019)
(0) vein tubular through the whole length (not counting the last 10% of the vein), same colour as or slightly lighter than the colour of the surrounding veins;
(1) vein partly spectral (not counting the last 10% of the vein);
(2) vein throught whole lenght spectral (+-); (3) absent

**#154 Hind wing, ratio of 1Rs to rs-m [ordered]:** (Klopfstein & Spasojevic, 2019)
Write ratio.

**#155 Basal hamuli, location:** (Klopfstein & Spasojevic, 2019)
(0) some distance from wing base, on membrane or on spectral vein;
(1) close to wing base, on spur of tubular vein;
(2) absent, though in their place often with slightly thickened hairs;
(3) very large number of basal (!) hamuli along entire margin, half grown over by a more sclerotized narrow wing membrane in front of Sc + R (Hybrizontinae).

**#156 Basal hamuli, number:** (Klopfstein & Spasojevic, 2019)
(0) one;
(1) two;
(2) three;
(3) four;
(4); eight.

**#157 Distal hamuli, number: (Alvarado Gutiérrez, 2018)**(0) seven to three;
(1) eight or more.

**#158 Main body colour ("ground plan colour")** (Klopfstein & Spasojevic, 2019)
(0) black or brown;
(1) orange;
(2) yellow.

**#159 Face: main colour** (Klopfstein & Spasojevic, 2019)
(0) same as ground plan colour in both sexes;
(1) same as ground plan colour in female, male with different colour;
(2) same as ground plan colour in male, female with different colour;
(3) different than ground plan colour in both sexes.

**#160 Metasoma, shape [ordered]:** (Klopfstein & Spasojevic, 2019)
(0) depressed to cylindrical all the way;
(1) compressed from segment 5;
(2) compressed from segment 4;
(3) compressed from segment 3.

**#161 First tergite, dorso-lateral longitudinal carina, location:** (Klopfstein & Spasojevic, 2019)
(0) more or less complete, above spiracle;
(1) more or less complete, below or at spiracle;
(2) more or less absent, at most a vestige anteriorly and/or posteriorly;
(3) more or less complete but far below spiracle, posteriorly abruptly curved upwards.

**#162 First tergite, glymma [ordered]:** (Klopfstein & Spasojevic, 2019)
(0) absent;
(1) present, but rather shallow, not meeting at midline (although each glymma may join lateral sides of anterodorsal sulcus of T1) (cf. Phytodietus);
(2) deep, almost meeting at midline (cf. Netelia).

**#163 First sternite, apically, fusion to T1 [ordered]:** (Klopfstein & Spasojevic, 2019)
0) not fused to T1 (cf. Rhyssa);
(1) fused, but suture visible (cf. Megarhyssa);
(2) fused, no suture visible.

**#164 First tergite, dorsal view, dimension [ordered]:** (Klopfstein & Spasojevic, 2019)
(0) subquadrate;
(1) elongate, from about 1.5–3.5 times as long as posteriorly wide;
(2) very long and slender, >4.0 times as long as posteriorly broad.

**#165 First tergite, dorsal view, shape:** (Klopfstein & Spasojevic, 2019)
(0) petiolate, clear separation of postpetiolus;
(1) parallel sided;
(2) evenly tapering to front

**#166 First tergite, laterally, shape:** (Klopfstein & Spasojevic, 2019)
(0) rounded basally or with a hump around mid length, thus in profile more narrow in the front than in the apical half;
(1) flat in profile, at most with a very weak rounding basally, thus of even thickness in profile;
(2) basally straight and sometimes continuously expanding in profile, and with an angle in second half (often when petiolate).

**#167 First tergite, constriction:** (Klopfstein & Spasojevic, 2019)
(0) no diagonal constriction around mid length of tergite;
(1) with a constriction showing as a diagonal groove on the sides becoming a transverse impression dorsally (cf. Xorides).

**#168 Metasoma, separation of T1 and T2:** (Klopfstein & Spasojevic, 2019)
(0) separated by a normal joint, thus as flexible as T2 to T3;
(1) partially fused so that T2 cannot move independently (cf. Metopius).

**#169 Metasoma, T1, length of latero-median carinae [ordered]:** (Klopfstein & Spasojevic, 2019)
(0) absent;
(1) less than half length of tergite;
(2) more than half length of tergite.

**#170 Metasoma, T1, position of latero-median carinae:** (Klopfstein & Spasojevic, 2019)
N/A=absent or too short to tell;
(0) converging, but then still about as far apart as distance to lateral margin;
(1) very strongly converging, ending up very close together when they become parallel;
(2) just parallel, (on elongate T1).

**#171 Metasoma, T1, position of spiracle:** (Klopfstein & Spasojevic, 2019)
(0) at or anterior to 0.6 times length of segment;
(1) posterior to 0.6 times length of segment.

**#172 Metasoma, T1, sculpture on dorsal surface in the posterior half:** (Klopfstein & Spasojevic, 2019)
(0) smooth and impunctate;
(1) finely punctate, punctures small and distant;
(2) very coarsely punctate, with punctures separated inter se by about their own diameter;
(3) coarsely and closely punctate, the punctures more or less contiguous (but no rugae);
(4) shagreened;
(5) with sharp transverse rugae more or less punctured inbetween;
(6) with longitudinal parallel dense rugae and indistinct punctures (see Ichneumon);
(7) rugulopuntured, rugae more or less longitudinal, interupted, with punctures inbetween;
(8) shagreened and longutudinal rugae

**#173 Metasoma, T1, laterotergite 1, shape:** (Klopfstein & Spasojevic, 2019)
(0) more or less absent;
(1) broad on almost entire length of tergite and membranous;
(2) short and triangular but, with at least some colour (cf. Orthocentrinae: Dialipsis);
(3) elongate triangular with some colour;
(4) triangular on almost entire lenght and membranous.

**#174 Metasoma, S1, length, compared with T1 [ordered]:** (Klopfstein & Spasojevic, 2019)
(0) very short, <0.3;
(1) about 0.4–0.5 of length of tergite;
(2) very long, >0.6 - 0.9;
(3) almost till the end of tergit.

**#175 Metasoma, sternite 1, shape:** (Klopfstein & Spasojevic, 2019)
(0) with a weak to strong median keel anteriorly;
(1) flat, without a median longitudinal keel;
(2) with a median anterior invagination;
(3) as 0 but with additionally transversal carinae behind

**#176 Metasoma, sternite 1, central ornamentation:** (split from Klopfstein & Spasojevic, 2019)
(0) absent;
(1) present.

**#177 Metasoma, sternite 1, central ornamentation, modification:** (split from Klopfstein & Spasojevic, 2019)
(0) with a low rounded swelling;
(1) with a strong sharp transverse crest;
(2) with a pointed tubercle;
(3) with paired tubercles;
(4) with large swelling;
(5) with a strong protruding longitudinal swelling (Hyperacmus);
(6) with a low round swelling and longitudinal crest (Allomacrus);
(7) with paired round swellings;
(8) longitudinal swelling on almost whole of the S1 length.

**#178 Metasoma, sternite 1, hind margin, shape:** (Klopfstein & Spasojevic, 2019)
(0) from transverse to shallowly V-shaped;
(1) sharply V-shaped with a median notch;
(2) with a very deep cleft, almost bifid;
(3) with a median posteriorly directed point.

**#179 Metasoma, T2, dorsal view, length [ordered]:** (Klopfstein & Spasojevic, 2019)
(0) transverse to subquadrate;
(1) 1.3–2.5 times as long as posteriorly broad;
(2) >2.5 times as long as posteriorly broad.

**#180 T2: latero-median carinae:** (Klopfstein & Spasojevic, 2019)
(0) absent;
(1) present as two parallel carinae;
(2) present, fused in a single medial carina.

**#181 Thyridium, presence:** (Klopfstein & Spasojevic, 2019)
(0) absent;
(1) present.

**#182 Thyridium and gastrocoelus, location:** (Klopfstein & Spasojevic, 2019)
(0) thyridium not sunken in gastrocoelus;
(1) with deep transverse depression ('gastrocoelus') in which is the thyridium (Figs 67, 68, 69);
(2) thyridium in impression surrounded by carinulae.

**#183 Thyridium, shape and position:** (Klopfstein & Spasojevic, 2019)
(0) present, ovoid, less than one length or diameter distant from anterior edge of T2;
(1)  present, ovoid, more than or equal to one length or diameter distant from anterior edge;
(2) present, tranverse, less than one length or diameter distant from anterior edge;
(3) present, tranverse, more than or equal to one length or diameter distant from anterior edge of T2.

**#184 Metasoma, T2, anterior half, impressions:** (modified from Klopfstein & Spasojevic, 2019)
(0) simple, with no impression behind thyridium (Fig. 67);
(1) with a shallow or deep, very oblique impression starting +- medially close to base and pointing towards spiracle or slightly basal to it, thus subtending an angle of >45° to longitudinal axis (Fig. 66);
(2) with a deep, oblique impression starting at base of tergite, about halfway between middle and lateral edge of tergite, and pointing a little behind spiracle, subtending an angle of <40° to longitudinal axis (Fig. 65);
(3) as state 2, but starting very close to middle of base of tergite;
(4) with a weak to moderately strong transverse impression, sometimes with this only present laterally and not meeting on mid-line;
(5) with both an oblique (as states 1-3) and a transverse impression (as 4), both meeting close to lateral edge of tergite;
(6) with oblique impressions starting a bit remote from base and meeting an equally strong, subapical transverse carina clearly before meeting lateral edge of tergite (cf. Lycorina).

**#185 Metasoma, T2, centrally, sculpture:** (Klopfstein & Spasojevic, 2019)
(0) smooth and polished, sometimes with a few isolated punctures (Fig. 68); (1) evenly shagreened and matt;
(2) with deep punctures (Fig. 69);
(3) transversely aciculate;
(4) rugopunctate;
(5) finely microreticulate;
(6) with longitudinal striae at least in basal part;
(7) finely evenly punctured.

**#186 Metasoma, T2, anterior margin medially in cross-section:** (Klopfstein & Spasojevic, 2019)
(0) broadly rounded;
(1) with a single, sharp, median longitudinal ridge;
(2) with a shallow, median longitudinal concavity (Gauld et 2002, Fig. 68);
(3) with two parallel carinae (which continue the lateromedian carinae of T1).

**#187 Metasoma, T2 (and usually T3/T4), sculpture on posterior 0.2:** (Klopfstein & Spasojevic, 2019)
(0) not sculpturally differentiated from anterior part of tergite, at most with extreme posterior end of tergite impunctate;
(1) with sculpture differing from rest of tergite (Figs 65, 66, 69), usually smooth and impunctate.

**#188 Metasoma, laterotergite 2 [ordered]:** (Klopfstein & Spasojevic, 2019)
(0) more or less absent;
(1) moderately broad, 0.25–0.4 times as wide as long, and strongly sclerotized;
(2) broad, >0.5 times as wide as long, and membranous.

**#189 Laterotergite 2, crease:** (Klopfstein & Spasojevic, 2019)
(0) creased and curved mesad under metasoma (Fig. xx Netelia;
(1) not separated by a crease (Fig. xx Allophrys sp).

**#190 T2, spiracle position:** (Klopfstein & Spasojevic, 2019)
(0) on dorsal part or directly on crease;
(1) clearly below the crease.

**#191 T2 and T3, fusion:** (Klopfstein & Spasojevic, 2019)
(0) separate, with flexion line allowing movement (Fig. 2);
(1) fused, without a flexion line.

**#192 T3, surface and impressions:** (Klopfstein & Spasojevic, 2019)
(0) more or less evenly convex, without strongly raised areas;
(1) with conspicuous lateromedian rounded swellings (Fig. 71);
(2) with a median rhombic raised area (Fig. 72);
(3) with a transverse, lozengeshaped area, bordered by deep grooves, at least posteriorly;
(4) with diagonal grooves starting basally close to the middle and pointing towards posterior corners of tergite, although not always reaching them (cf. Banchinae - Glyptini);
(5) with lateromedian round swellings in basal half and subapical transverse impression, between which there is a basally pointing flat triangle outlined by strong grooves (cf. Lycorina).

**#193 T3: latero-median carinae: (Alvarado Gutiérrez, 2018)**(0) absent;
(1) present as two parallel carinae;
(2) present, fused in a single medial carina

**#194 Metasoma, laterotergite 3 [ordered]:** (Klopfstein & Spasojevic, 2019)
(0) more or less absent;
(1) moderately broad, 0.25–0.4 times as wide as long, and strongly sclerotized;
(2) broad, >0.5 times as wide as long, and membranous.

**#195 Laterotergite 3, crease:** (Klopfstein & Spasojevic, 2019)
(0) separated by a crease at least until the spiracle, posterior part can be without the creast and often turned under (Fig. xx);
(1) not separated by a crease (Fig. xx Allophrys).

**#196 T3-T4, posterolateral corners:** (Klopfstein & Spasojevic, 2019)
(0) more or less rounded or right-angled;
(1) incised, so corner is acute;
(2) obliquely truncate;
(3) tergites extended backwards laterally, so that they are shorter medially (cf. Phthorima, Campocraspedon in Diplazontinae).

**#197 T3-T4, colour pattern:** (Klopfstein & Spasojevic, 2019)
(0) uniformly colored or with a very narrow end margin;
(1) with color different only in the last 0.2 of the tergites;
(2) with colour different on more than 0.2 of the tergite
(3) with pattern in the form of paired spots or connected spots

**#198 Metasoma, laterotergite 4 [ordered]:** (Klopfstein & Spasojevic, 2019)
(0) more or less absent;
(1) moderately broad, 0.25–0.4 times as wide as long, and strongly sclerotized;
(2) broad, >0.5 times as wide as long, and membranous.

**#199 Metasoma, T7, size, compared to tergite 6, female [ordered]:** (Klopfstein & Spasojevic, 2019) (0) conspicuously shorter (at most 0.6x as long as tergite 6);
(1) similar in size to tergite 6, sometimes partially retraced under it, but not very noticeably smaller; (2) conspicuously longer than tergite 6.

**#200 Metasoma, T7, size, compared to tergite 6, male [ordered]:** (Klopfstein & Spasojevic, 2019)
(0) conspicuously shorter (ab most 0.6x as long as tergite 6), or retracted invisible;
(1) of similar size to or slightly smaller than tergite 6;
(2) longer and usually more strongly sclerotized than tergite 6.

**#201 Laterotergite 4, crease:** (Klopfstein & Spasojevic, 2019)
(0) separated by a crease (at least basally), often turned under;
(1) not separated by a crease (Fig. xx Allophrys).

**#202 S7: sclerotization in male:** (Klopfstein & Spasojevic, 2019)
(0) flat and evenly sclerotized;
(1) weakly sclerotized with a median longitudinal ridge.

**#203 Anterior sternites of female (usually at least S2-S4):** (Klopfstein & Spasojevic, 2019)
(0) without ovipositor guides;
(1) with tubercular ovipositor guides (Fig. xx Megarhyssa).

**#204 Tergite 8, lower anterolateral corner, female:** (Klopfstein & Spasojevic, 2019)
(0) with a small pointed or finger like apodeme (Figs 95, 96, 97);
(1) with apodeme enlarged (Fig. 94).

**#205 Tergite 8, size and shape, female:** (modified from Klopfstein & Spasojevic, 2019)
(0) short;
(1) elongate dorsally, but not forming a horn or boss (Fig xx Odontocolon albotibial);
(2) elongate dorsally into a horn or boss (Fig. xx Megarhyssa g. greenei);
(3) laterally elongate, dorsally with a hole through which T9 is visible (see Xenoschesis, Ctenopelmatinae);
(4) T9 very short along mid-line, lateral portions expanding further backwards.

**#206 T9 in female:** (Klopfstein & Spasojevic, 2019)
(0) from triangular to strap-like, immutably fused with preceding tergite (though the join is generally discernible because of differing sculpture patterns) and occupying an indentation in it;
(1) almost D-shaped, and positioned behind (rather than within) T8, the joint between the two very weakly sclerotized;
(2) separated (some outgroup);
(3) forming a cornus (some outgroups);
(4) within the cavity of T8 (cf. Xenoschesis)

**#207 T9, division in male:** (Klopfstein & Spasojevic, 2019)
(0) entire;
(1) medially longitudinally divided.

**#208 T8-T9, separation from between tergites in male:** (Klopfstein & Spasojevic, 2019)
(0) laterally separated, with tergite VIII forming a lateral plate on the side of tergite IX (Fig. 99);
(1) with tergites VIII and IX completely fused laterally, seemingly just a single tergite (Figs 100, 101).

**#209 S8, shape in male:** (modified from Klopfstein & Spasojevic, 2019)
(0) more or less transverse and moderately sclerotized (Fig. 78);
(1) strongly sclerotized, short, deeply convex, closing apex of metasoma at rest (Figs 79, 80);
(2) weakly to moderately sclerotized and rather flat, but elongate, more or less closing apex of metasoma at rest (Figs 81, 82);
(3) similar to (0), but with a median emargination (very small one, so maybe it should go to 0);
(4) narrow, paddle-shaped (cf. some Diplazontinae);
(5) strongly and evenly concave posteriorly.

**#210 Apex of aedeagus:** (Klopfstein & Spasojevic, 2019)
(0) subcylindrical;
(1) dorsoventrally depressed.

**#211 Parameres, size:** (modified from Klopfstein & Spasojevic, 2019)
(0) about 1-2x as long as wide at mid length;
(1) very strongly elongate, can also be rod-like apically (cf. Mesochorus);
(2) very short, inconspicous.

**#212 Sternite 6, female (hypopygium), shape:** (Klopfstein & Spasojevic, 2019)
(0) transverse (wider than long when viewed from below), with posterior margin simple and more or less straight (not convex), in profile inconspicuous or barely visible (e.g. Pimplini);
(1) transverse, with posterior margin simple and more or less straight (not convex), in profile square or rectangle, more conspicuous (e.g. Campopleginae and probably most of the other groups with strongly compressed metasoma);
(2) elongate (longer than wide), posterior margin simple and convex, in profile small, more or less triangular but with the longest side rounded (e.g. Clistopyga);
(3) viewed from below as wide as long or slightly elongate, posterior margin simple and slightly convex, in profile forming equilateral triangle or if scalene triangle, then the longest side straight (e.g. Mesochorinae);
(4) sub-quadrate to elongate, posterior margin convex with median notch (cut), in profile diverse with lower margin simply straight or broken at the place where the notch starts (e.g Banchinae);
(5) moderately long, regularly triangular in profile, medially membranous (e.g. Lycorina);
(6) very elongate with lateral sides strongly converging towards the end, posterior margin angeled with notch (cut) present and sometimes extending all the way to the base, in profile elongate and strongly triangular (e.g. Acaenitinae);
(7) transverse with a clift medialy where ovipositor passes

**#213 Ovipositor sheath, pubescence:** (Klopfstein & Spasojevic, 2019)
(0) dense and short (longest hairs shorter than the width of ovipositor sheath);
(1) dense and long (longest hairs exceeding width of sheath);
(2) sparse and long (hairs separated by several times their diameter, and longer than width of sheath);
(3) sparse and short;
(4) nearly hairless, only hairs are along the inner lower margin;
(5) nearly hairless, only some long sparse hairs on appical half.

**#214 Ovipositor sheath, shape:** (modified from Klopfstein & Spasojevic, 2019)
(0) more or less parallel sided, sometimes continuously enlarged towards apex or clearly enlarged at tip (cf. Apechthis);
(1) distinctly enlarged around middle, more narrow before and after (cf. Thymaris, Zagryphus etc. in Tryphoninae);
(2) evenly narrowed at least on posterior half;
(3) broad until close to the end, then narrowed, along most of the length with lower part thickened to narrow the interior channel (seen on the inside of the sheats - cf. Astiphromma).

**#215 Ovipositor sheath, length compared to metasoma length [ordered]:**Ratio ovipositor sheaths to metasoma length.

**#216 Ovipositor, lateral view, shape:** (modified from Klopfstein & Spasojevic, 2019)
(0) parallel sided;
(1) evenly tapered;
(2) evenly tapered with enlarged submedian region of lower valve (Fig. 76);
(3) lower valve widened twice, in front and after notch in dorsal valve (Diplazontinae);
(4) lower valves swollen from about basal 0.2 to apical 0.2, swelling both down and towards lateral, thus broader than upper valve on most of length.

**#217 Ovipositor, tip, shape:** (Klopfstein & Spasojevic, 2019)
(0) more or less straight;
(1) up-curved;
(2) bowed downwards along at least posterior half;
(3) sinuous;
(4) abruptly decurved (bent down) only close to the apex.

**#218 Ovipositor, base ventrally:** (Klopfstein & Spasojevic, 2019)
(0) simple;
(1) swollen (Figs 76, 77).

**#219 Ovipositor, sculpture:** (Klopfstein & Spasojevic, 2019)
(0) smooth, apparently unsculptured;
(1) matte;
(2) at least lower valve weakly to strongly wrinkled.

**#220 Ovipositor, transection [ordered]:** (Klopfstein & Spasojevic, 2019)
(0) strongly compressed (Fig. 74);
(1) subcylindrical, or weakly compressed (Fig. 73);
(2) depressed.

**#221 Ovipositor, apical region, flexibility:** (Klopfstein & Spasojevic, 2019)
(0) rigid;
(1) capable of being deformed so extreme apex is deflected downwards.

**#222 Ovipositor, lower valves, apex, teeth:** (Klopfstein & Spasojevic, 2019)
(0) with distinctly oblique moderately interspaced teeth;
(1) without discernible teeth;
(2) with moderately interspaced teeth (except the most proximal one or two) vertical;
(3) with fine file-like teeth.

**#223 Ovipositor, lower valves, shape of basal tooth:** (Klopfstein & Spasojevic, 2019)
(NA) no teeth visible on lower valve;
(0) basal tooth unspecialized, similar to following teeth;
(1) basal tooth enlarged and forming barb.

**#224 Ovipositor, basal to apex lower valves, scabrous areas:** (Klopfstein & Spasojevic, 2019)
(0) without discrete scabrous areas;
(1) with one or more small scabrous areas.

**#225 Ovipositor, lower valve, lobe [ordered]:** (Klopfstein & Spasojevic, 2019)
(0) not enclosing the upper (Figs 73, 74);
(1) with a clear lobe partially enclosing upper valve (Fig. 75);
(2) apically entirely enclosing the upper.

**#226 Ovipositor, dorsal valve, apex, modifications:** (Klopfstein & Spasojevic, 2019)
(0) smooth, simply tapered, with or without nodus;
(1) with lateral row of small denticles;
(2) with a dorsal row of low teeth;
(3) with dorsal, subapical notch (Fig.Wahl Fig. 4.01 Exetastes);
(4) slender and needle like (Wahl Fig. 1-33 Astiphromma);
(5) weakly sclerotized (Euceros);
(6) slender, simply tapered (similar to 4 but polysphincini state);
(7) as state 3 but expanded laterally just before the notch;
(8) with two high teeth/nodi (Certonotus);
(9) with two tubercules (Xorides).
