# Supplementary material for: Impact of increasing morphological information by micro-CT scanning on the phylogenetic placement of Darwin wasps (Hymenoptera, Ichneumonidae) in amber
Source: Swiss J Palaeontol. 2023 Nov 3;142(1):30. doi: 10.1186/s13358-023-00294-2 (PMC10624732; doi:10.1186/s13358-023-00294-2)
Supplement: Supplementary file 3 — Additional file 3. Taxon and character sampling of the data set. [file 13358_2023_294_MOESM3_ESM.docx]

**Candidate subfamilies**

The candidate subfamilies for our fossils are as followed: Metopiinae (for #DO_3441M), Rhyssinae (for #NHMB_2811), Stem-Ophioniformes or Pimplinae (for #NHMD_876111) and Phygadeuontinae or Cryptinae (for #NHMD_876130). Two of the candidate subfamilies (Rhyssinae, Pimplinae) were already sufficiently sampled in Spasojevic et al. (2021). We extended the extant taxon sampling of the candidate subfamilies that were underrepresented by additional 37 extant taxa. We also added eight additional fossil representatives of the candidate subfamilies to analyse potential resemblance to the newly described fossils (see Supplementary file 1 for the taxon sampling). In order to reduce the complexity of the phylogenetic analyses, we reduced the number of taxa belonging to non-target subfamilies that were heavily sampled in the original matrix. In total, we included 172 extant and 27 fossil specimens, including the four newly described species.

Thirteen characters were excluded from Spasojevic et al. (2021) when they were never visible in the fossil specimens and were not significant for grouping any of the clades of interest. Six characters were split into two characters, one binary (absent/present) and one multistate. We further extended the matrix with several new states and 12 new characters, including some characters from previous publications to further distinguish various taxonomic groups (Alvarado, 2018; Broad et al., 2018; Meier et al., 2022) (see Supplementary file 2 for character description).

**References:**

Alvarado, M. (2018). Phylogeny of the wasp subfamily Metopiinae and patterns of speciation in the Exochus albiceps species-group Phylogeny of the wasp subfamily Metopiinae and patterns of speciation in the Exochus albiceps species-group (Issue July) [PhD Thesis, Dissertation, University of Kansas]. https://kuscholarworks.ku.edu/handle/1808/27984

Broad, G. R., Shaw, M. R., & Fitton, M. G. (2018). Ichneumonid wasps (Hymenoptera: Ichneumonidae): Their classification and biology. In Handbooks for the Identification of British Insects (Vol. 7).

Meier, N., Wacker, A., & Klopfstein, S. (2022). New fossil wasp species from the earliest Eocene Fur Formation has its closest relatives in late Eocene ambers (Hymenoptera, Ichneumonidae, Pherhombinae). Bulletin of the Society of Systematic Biologists, 1(1). https://doi.org/10.18061/bssb.v1i1.8427

Spasojevic, T., Broad, G. R., Sääksjärvi, I. E., Schwarz, M., Ito, M., Korenko, S., & Klopfstein, S. (2021). Mind the Outgroup and Bare Branches in Total-Evidence Dating: A Case Study of Pimpliform Darwin Wasps (Hymenoptera, Ichneumonidae). Systematic Biology, 70(2), 322–339. https://doi.org/10.1093/sysbio/syaa079
