# Supplementary material for: Impact of increasing morphological information by micro-CT scanning on the phylogenetic placement of Darwin wasps (Hymenoptera, Ichneumonidae) in amber
Source: Swiss J Palaeontol. 2023 Nov 3;142(1):30. doi: 10.1186/s13358-023-00294-2 (PMC10624732; doi:10.1186/s13358-023-00294-2)
Supplement: Supplementary file 5 — Additional file 5. Molecular sampling of additional taxa, and PCR conditions. [file 13358_2023_294_MOESM5_ESM.docx]

**Methods for molecular data**

The new Metopiinae material was either from the Swedish Malaise Trap Project (SMTP, Sweden), the Natural History Museum St. Gallen (NMSG, StGallen, Switzerland) or the Natural History Museum in Basel (NMBS, Switzerland). The specimens were either stored in 95% Ethanol (SMTP material) or dried (NMBS, NMSG).

We sequenced three genes (28S, COI, CAD) from 10 additional Metopiinae specimens from tissue samples using standard protocols.DNA extraction from leg tissue took place at the University of Basel (Evolutionary Biology, Salzburger Lab) using the DNeasy blood and tissue kit with standard protocol (Qiagen). We modified the protocol so that we prolonged the digestion step to overnight at 56 C° and did two elusion steps with 50 ml each. All the PCR conditions (primers and annealing temperatures) are reported in a table below. We checked the quality of the PCR product on a 1% Agarose gel before sending the PCR products w for clean-up and sequencing to Macrogen Europe in the Netherlands. The sequences were edited and aligned in Geneious (Geneious Prime 2023.0.1) with the MUSCLE alignment algorithm using default settings. The final sequences are deposited in GenBank (see additional file 1 for accession numbers).

**PCR conditions**

|  | Time | Temperatur in °C for CO1, and CO1 short | Cycles |
| --- | --- | --- | --- |
| Initial denaturation | 2min | 94 | 1 |
| Annealing 1 | 30s | 54 | 2 |
| Annealing 2 | 30s | 52 | 2 |
| Extention | 1min | 72 | 2 |
| Denaturation | 30s | 94 | 35 |
| Annealing final | 30s | 50 | 35 |
| Extention | 1min | 72 | 35 |
| Extention final | 10min | 72 | 1 |
|  |  |  |  |
|  |  |  |  |
|  | Time | Temperatur in °C for 28S | Cycles |
| Initial denaturation | 2min | 94 | 1 |
| Annealing 1 | 30s | 56 | 2 |
| Annealing 2 | 30s | 54 | 2 |
| Extention | 1min | 72 | 2 |
| Denaturation | 30s | 94 | 35 |
| Annealing final | 30s | 52 | 35 |
| Extention | 1min | 72 | 35 |
| Extention final | 10min | 72 | 1 |
|  |  |  |  |
|  | Time | Temperatur in °C for CAD | Cycles |
| Initial denaturation | 2min | 94 | 1 |
| Annealing 1 | 30s | 56 | 2 |
| Annealing 2 | 30s | 54 | 2 |
| Extention | 1min | 72 | 2 |
| Denaturation | 30s | 94 | 35 |
| Annealing final | 30s | 52 | 35 |
| Extention | 1min | 72 | 35 |
| Extention final | 10min | 72 | 1 |
