# Supplementary figures and images for: Impact of increasing morphological information by micro-CT scanning on the phylogenetic placement of Darwin wasps (Hymenoptera, Ichneumonidae) in amber
Source: Swiss J Palaeontol. 2023 Nov 3;142(1):30. doi: 10.1186/s13358-023-00294-2 (PMC10624732; doi:10.1186/s13358-023-00294-2)

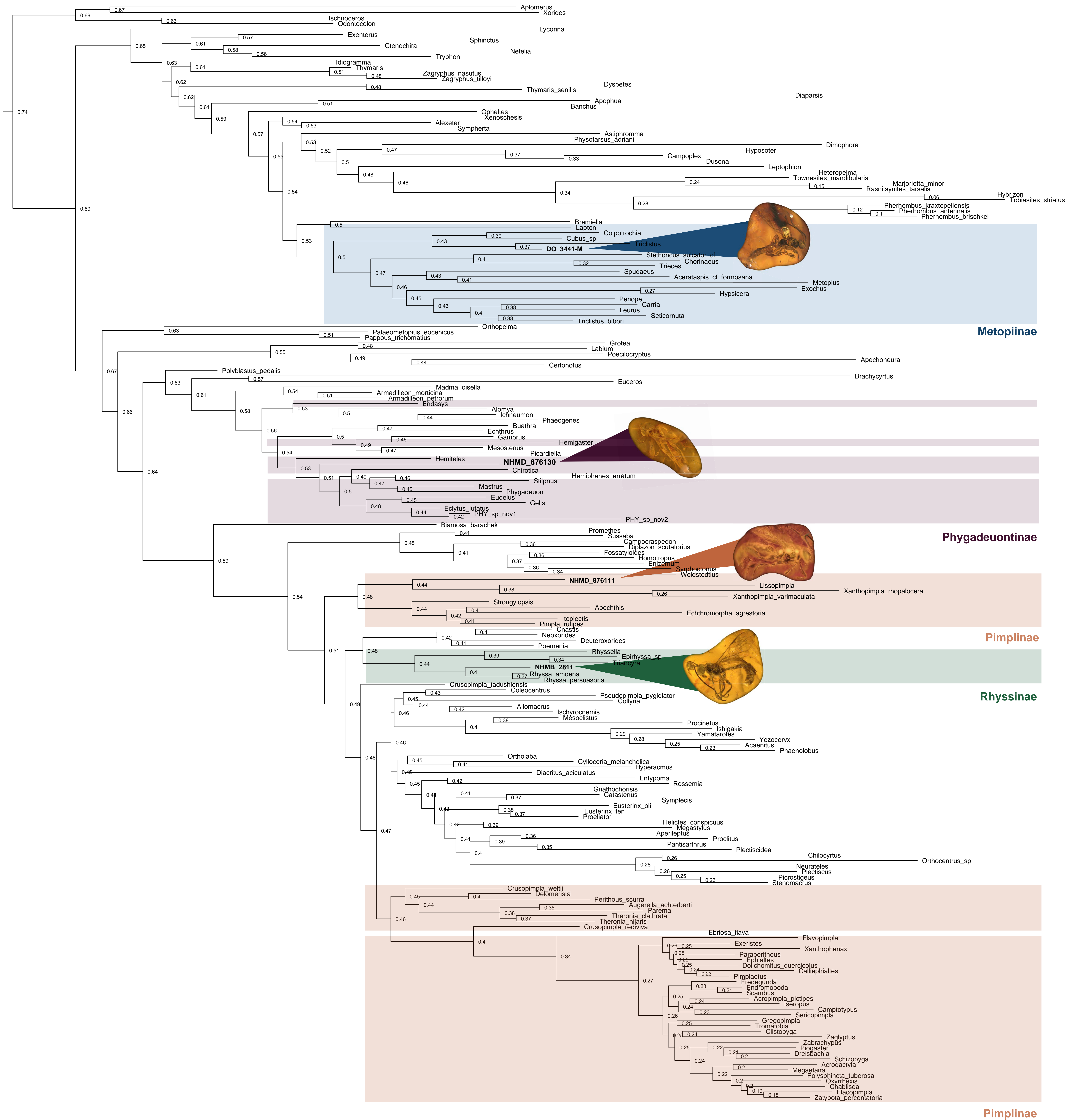

Supplement: Supplementary file 6 — Additional file 6. A majority rule consensus tree with all compatible groups added and with all fossils from the combined evidence analysis after the scan. [file 13358_2023_294_MOESM6_ESM.pdf]

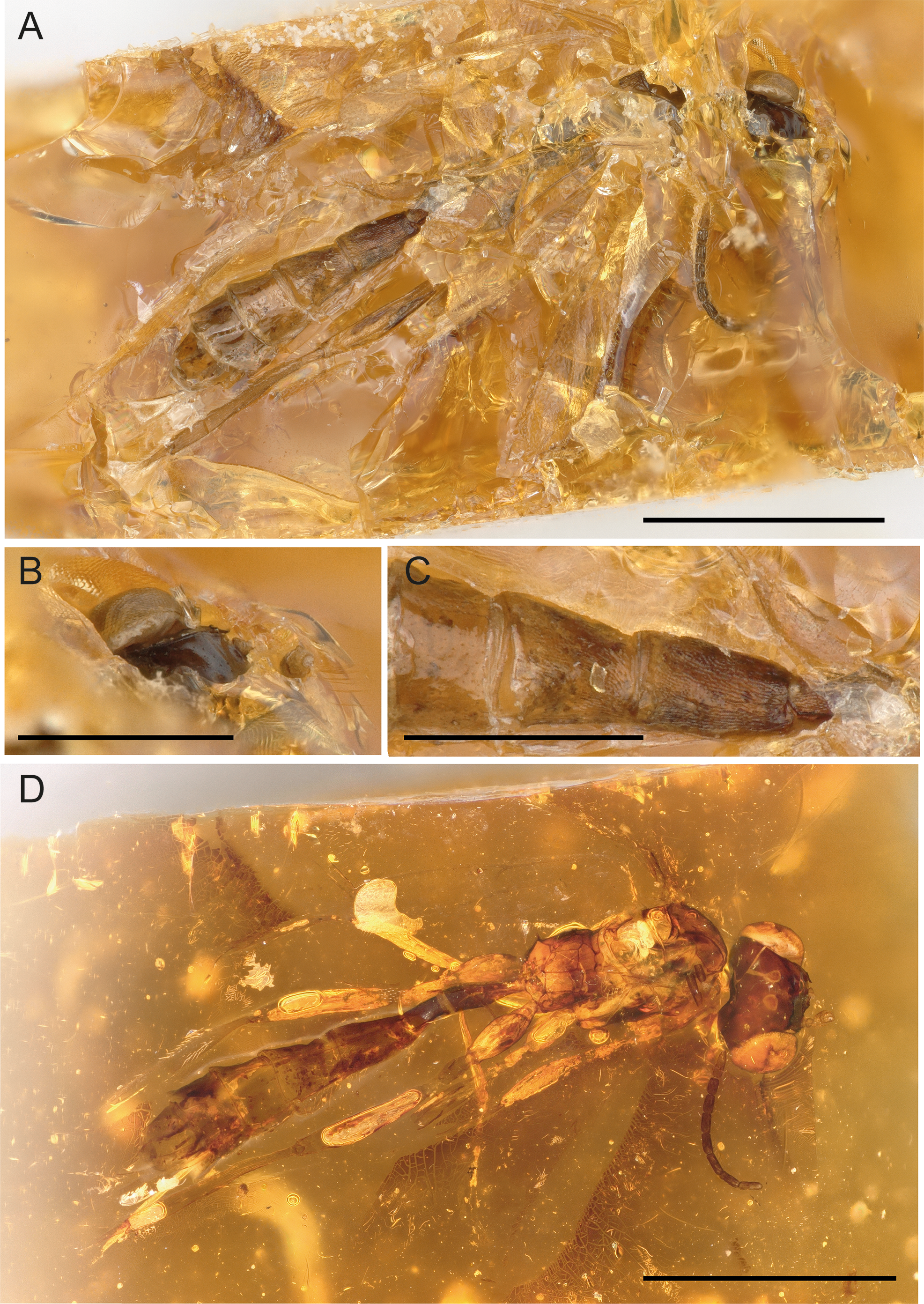

Supplement: Supplementary file 11 — Additional file 11. Additional photos of Magnocula sarcophaga gen. et sp. nov. after destruction. A Habitus of broken specimen in dorsal view. B Revealed head with eye. C Revealed partial metasoma with visible structures on T2-T4. D Habitus after epoxy cast to stabilize specimen in dorsal view. Scale bar: A: 1mm, B: 0.5mm, C: 0.5mm, D: 1mm. [file 13358_2023_294_MOESM11_ESM.png]
